# Context-Dependent Heterotypic Assemblies of Intrinsically Disordered Peptides

**DOI:** 10.1021/jacs.4c12150

**Published:** 2025-01-14

**Authors:** Yuchen Qiao, Ayisha Zia, Grace Wu, Zhiyu Liu, Jiaqi Guo, Matthew Chu, Hongjian He, Fengbin Wang, Bing Xu

**Affiliations:** † Department of Chemistry, Brandeis University, 415 South St., Waltham, Massachusetts 02454, United States; ‡ Department of Biochemistry and Molecular Genetics, University of Alabama at Birmingham, Birmingham, Alabama 35233, United States

## Abstract

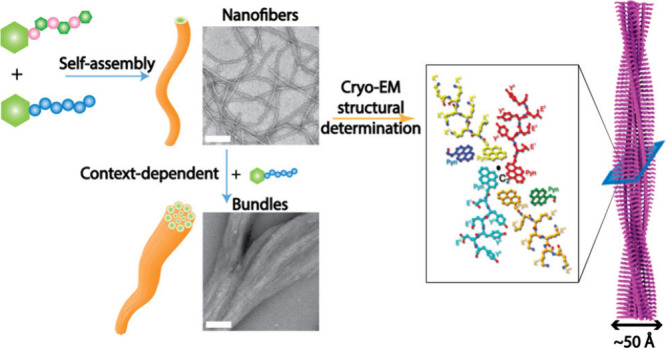

Despite their critical role in context-dependent interactions for protein functions, intrinsically disordered regions (IDRs) are often overlooked for designing peptide assemblies. Here, we exploit IDRs to enable context-dependent heterotypic assemblies of intrinsically disordered peptides, where “context-dependent” refers to assembly behavior driven by interactions with other molecules. By attaching an aromatic segment to oppositely charged intrinsically disordered peptides, we achieve a nanofiber formation. Although the same-charged peptides cannot self-assemble, oppositely charged peptides form heterotypic nanofibers. Cryo-EM analysis reveals a β-sheet arrangement within the ordered core of these nanofibers, conformational heterogeneity, and a disorder-to-order continuum and shows a high number of hydrogen bonds between tyrosine and lysine ε-amine. Additionally, this work demonstrates a post-assembly morphological change resulting from local conformational flexibility. While equal molar mixtures of the charged intrinsically disordered peptides yield nanofibers, doubling the positively charged peptides after assembly produces bundles of nanofibers. Furthermore, reducing the number of aromatic amino acid residues reduces bundle formation. Demonstrating context-dependent self-assembly of intrinsically disordered peptides and revealing atomistic insights into heterotypic assemblies of intrinsically disordered peptides for the first time, this work illustrates a straightforward approach to enable heterotypic intrinsically disordered peptides to self-assemble for the design of adaptive, multifunctional peptide nanomaterials.

This Communication reports the first cryo-EM structure of heterotypic assemblies formed by intrinsically disordered peptides and demonstrates their context-dependent assembly. Traditionally, peptide materials have used structured protein domains (α-helix and β-sheet) for specific functions^1−10^ like fibers and hydrogels in tissue engineering,^10,11^ drug delivery,^12,13^ and molecular electronics.^14^ However, a crucial aspect of proteins—intrinsically disordered regions (IDRs)^15^—has been largely overlooked. Despite lacking a fixed structure, IDRs undergo context-dependent conformational selection^16^ to bind with other proteins or form higher-order assemblies, making their binding and assembly behaviors highly adaptable to the specific molecular environment.^17^ Like structured domains, IDRs are essential protein building blocks with a critical role in regulating cellular processes.^15^ For instance, enriched IDRs in human transcription factors^18,19^ aid in recognizing specific DNA sequences.^20^ Additionally, IDRs flank kinase domains for signal regulation,^21^ play a role in the dispersal and movement of membrane contact site proteins,^22,23^ control cBAF activities,^23^ and contribute to the formation of ribonucleoprotein (RNP) granules.^24^ Notably, IDRs are also found in pathological proteins associated with neurodegenerative diseases.^23,25^

Focusing only on structured domains for peptide assembly design overlooks the potential of IDRs, whose flexibility enables novel functionalities. The flexibility allows intrinsically disordered peptides to interact diversely with other molecules (heterotypic interactions). By harnessing these context-dependent characteristics—where assembly behavior depends on both intrinsic structure and molecular environment—we can develop responsive peptide-based nanomaterials. Though interest in intrinsically disordered peptide assemblies is growing,^26−32^ several critical questions remain, such as how inherent flexibility permits diverse heterotypic interactions, the atomistic structures within heterotypic intrinsically disordered peptide assemblies, and whether conformational flexibility contributes to morphological variation. Addressing these fundamental questions is crucial for developing the next generation of peptide biomaterials that mimic proteins containing IDRs, like those in cytoskeletons,^33^ extracellular matrices,^34^ and biomineralization.^35^

Motivated by the above rationales, we investigated heterotypic assemblies of intrinsically disordered peptides with opposite charges. Pyrene, an aromatic segment, was appended to both positively and negatively charged peptides (Scheme 1). Same-charged peptides, even with pyrene, did not form homotypic assemblies. However, mixing equal molar oppositely charged peptides creates heterotypic nanofibers. Cryo-EM revealed a β-sheet arrangement within the self-assembled nanofibers with a C_2_ symmetry. Notably, one-third peptide segments within the nanofibers remain disordered, indicating conformational heterogeneity and disorder-to-order continuum. It reveals a high number of hydrogen bonds between tyrosine and lysine ε-amines near the p*K*_a_ of lysine. Additionally, this work shows a post-assembly morphological change resulting from local conformational flexibility—that is, doubling the positively charged peptides after assembly favored the formation of bundled nanofibers. Furthermore, reducing the number of aromatic amino acid residues decreases bundle formation. This work sheds light on context-dependent intrinsically disordered peptide interactions and offers insights for intrinsically disordered peptide self-assembly in developing adaptive, multifunctional peptide nanomaterials.

**Scheme 1:**
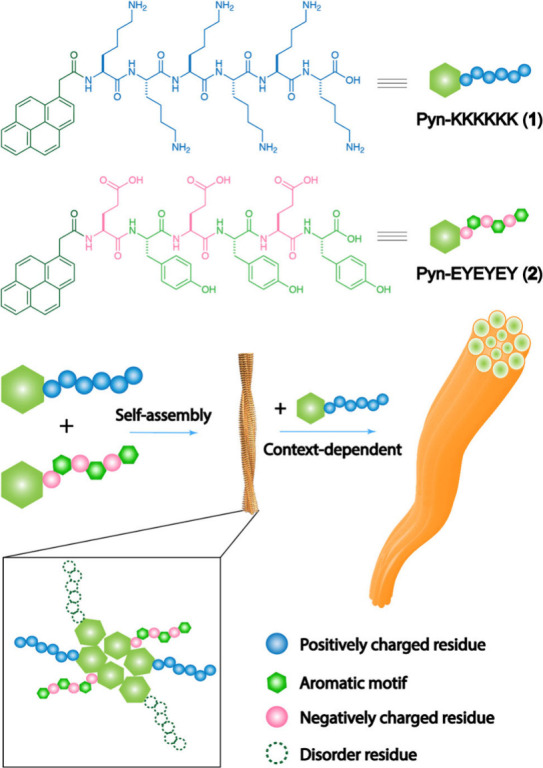
Two Oppositely Charged Intrinsically Disordered Peptides Self-Assemble To Form Nanofibers, Which Turn into Bundles via a Post-Assembly Control Disordered residues are depicted as dashed, hollow circles.

We selected KKKKKK as the positively charged intrinsically disordered peptide, a sequence found as a disordered region in proteins^36−38^ like KRas.^36^ We selected EYEYEY as the main negatively charged peptide for interacting with KKKKKK due to its presence in human proteins, such as MAP3K5, albeit without an experimentally verified ordered structure. This choice was informed by both bioinspiration, given the role of poly-EY motifs as kinase substrates, and rational design for studying context-dependent interactions with KKKKKK. To investigate tyrosine’s role, we increased the negative charges by replacing tyrosine with glutamic acid, creating sequences EEEYEY, EEEEEY, and EEEEEE (Scheme S1). EEEYEY is an IDR in transcription elongation factor SPT6,^39−41^ or SPT5.^42^ EEEEEY occurs in SPT5,^42^ pre-rRNA-processing protein TSR1 homologue,^29^ or malectin.^43^ EEEEEE is in a disordered region in proteins like E3-Ubiqutin ligase,^44^ huntingtin,^45^ tau-tubulin kinase 1,^46^ or AT-rich interactive domain-containing protein 1A.^47^ Conjugating these peptides to pyrene would generate peptides **1**–**5**. We chose pyrene as the N-terminal aromatic segment due to its fluorescence change, which allows easy monitoring of self-assembly.^30^ Peptides **1**–**5** were synthesized via solid-phase peptide synthesis,^48^ yielding milligram scale of each intrinsically disordered peptide with excellent yields (Figure S12).

We used pyrene fluorescence to determine the critical micelle concentrations (CMCs) of all intrinsically disordered peptides, finding values between 300 to 500 μM (Figures S2 and S3), exhibiting minimal variation (Figure 1A) and indicating their solubility in water. Notably, Pyn-KKKKKK (**1**), with pyrene attached to six lysines, has the highest CMC, while Pyn-EYEYEY (**2**), containing three tyrosine residues, has the lowest, consistent with their charged states at neutral pH. Replacing tyrosine with glutamic acid in Pyn-EEEYEY (**3**) raises its CMC slightly over Pyn-EEEEEY (**4**), while Pyn-EEEEEE (**5**) has a CMC lower than **1** but higher than **2**.

**Figure 1:**
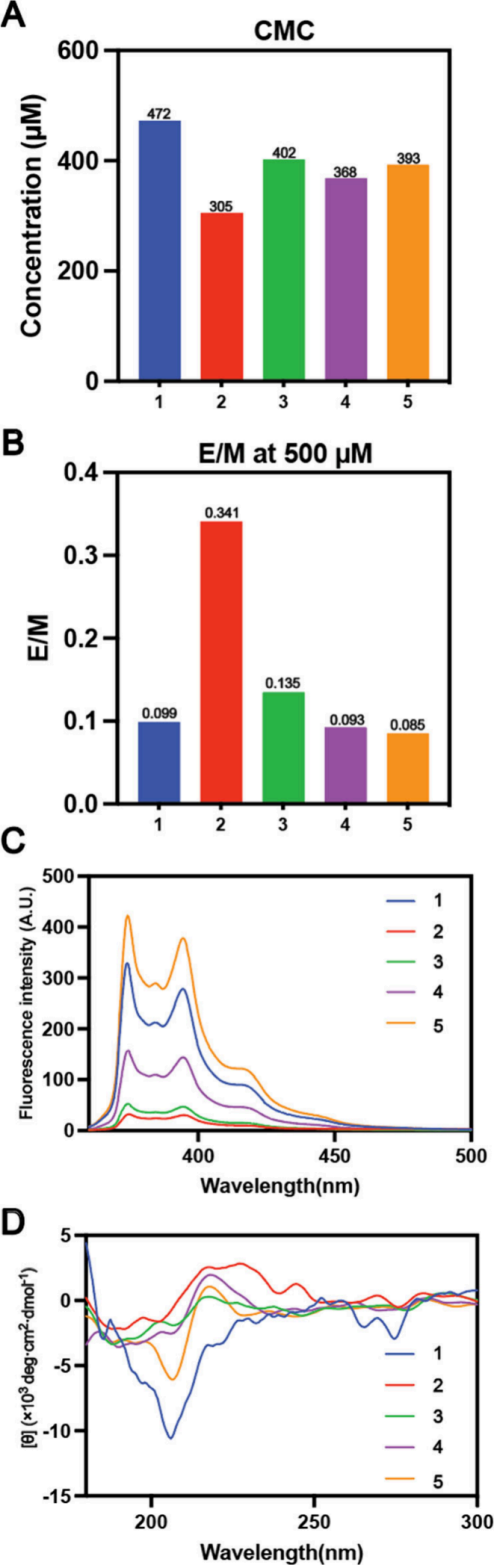
(A) CMCs of **1**–**5**. (B) Excimer/monomer ratio (E/M), (C) fluorescent spectra, and (D) CD spectra of **1**–**5** at 500 μM.

Using the excimer-to-monomer (E/M) fluorescence ratio at 500 μM, which is near their CMCs (Figure 1B), we estimated the relative quantity of excimers over monomers (Figure S2F). Pyn-EYEYEY (**2**) has the highest excimer quantity (E/M = 0.341), followed by Pyn-EEEYEY (**3**) (E/M = 0.14) and Pyn-EEEEEY (**4**) (E/M = 0.093). Compounds lacking tyrosine, such as Pyn-KKKKKK (**1**) and Pyn-EEEEEE (**5**), have E/M values below 0.1, indicating that tyrosine contributes to pyrene excimer formation. None of the peptides at 500 μM had an E/M value above 0.5, indicating monomer dominance at this concentration. Due to tyrosine’s hydroxyl group quenching pyrene fluorescence, peptides with tyrosine showed lower intensities (Figure 1C). At 500 μM, Pyn-KKKKKK (**1**) and Pyn-EEEEEE (**5**), which lack tyrosine, displayed intensities of 300–400 a.u. In contrast, Pyn-EEEEEY (**4**) with one tyrosine had a reduced intensity of ∼150 a.u., while Pyn-EEEYEY (**3**) with two tyrosines dropped below 100 a.u. Pyn-EYEYEY (**2**), containing three tyrosines, showed the lowest fluorescence intensity.

To examine the secondary structures of **1**–**5**, we conducted circular dichroism (CD) spectroscopy at 500 μM (Figure 1D). Compound **1** showed a broad negative peak from 190 to 220 nm and a sharp peak at 205 nm, indicating a disordered structure. Compound **5** also displayed a negative peak at 205 nm followed by a positive peak at 217 nm, suggesting a disordered structure. Compounds **2**, **3**, and **4** exhibited a broad positive peak from 200 to 240 nm, suggesting minimal β-sheet character, though the low signal intensity prevents conclusive secondary structure determination.

In peptide mixtures, adding Pyn-KKKKKK (**1**) to Pyn-EYEYEY (**2**) decreases the monomer peak and increases the excimer peak, suggesting an association between the two components (Figure 2A). Adding an equivalent amount of **1** further decreases the fluorescence signal, while 2 equiv reduces the excimer band from 430 nm to 600 nm and increases monomer peaks at 390 nm and 420 nm. In mixtures of other negatively charged intrinsically disordered peptides, the pyrene monomer peaks at ∼370 nm decrease as **1** is added, with excimer peak increases varying across compounds **3**, **4**, and **5**; and compounds **3** and **5** notably enhance the excimer peak at 480 nm (Figure S4).

**Figure 2:**
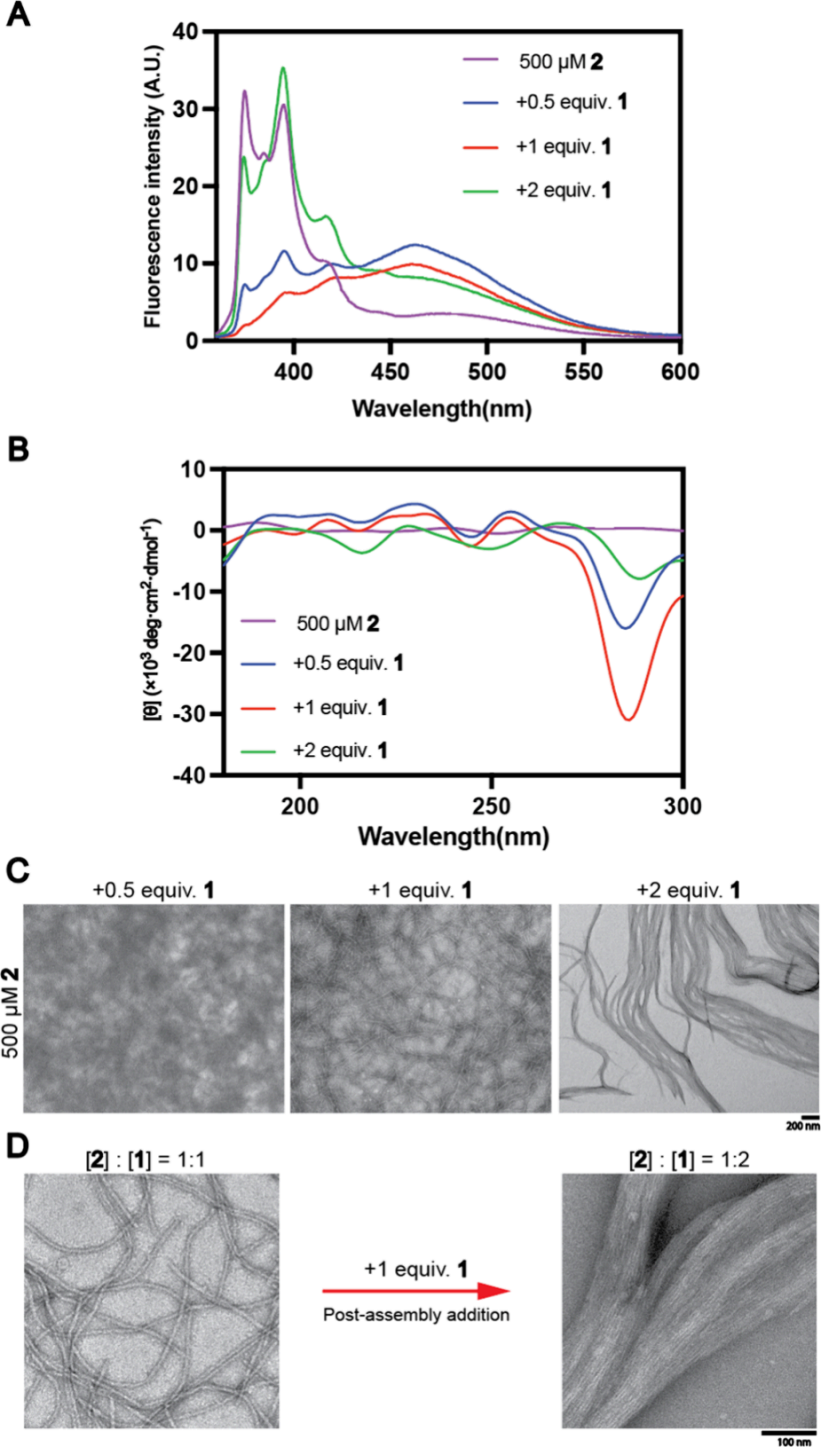
(A) Fluorescent and (B) CD spectra and (C) TEM images of **2** (500 μM) and its mixture with 0.5, 1, and 2 equiv of **1**. (D) Fiber-to-bundle transformation by adding 1 equiv of **1** to an equimolar mix of **1** and **2**.

CD spectra revealed trends in these mixtures (Figure 2B). Pyn-EYEYEY (**2**) alone shows no clear tyrosine CD signal, but adding **1** significantly induce a negative peak at 270–290 nm, characteristic of ordered l-tyrosine residues, which is consistent with the cryo-EM structure (Figure 3). The characteristic peak decreases with half equivalent and further reduces at two equiv, suggesting that adding excess positively charged peptide prompts aggregation and signal loss from the peptides in solution. Analogs with fewer tyrosine residues (**3**, **4** and **5**) lack a strong tyrosine peak at around 280 nm, with less than 5 units remaining (Figure S5).

**Figure 3:**
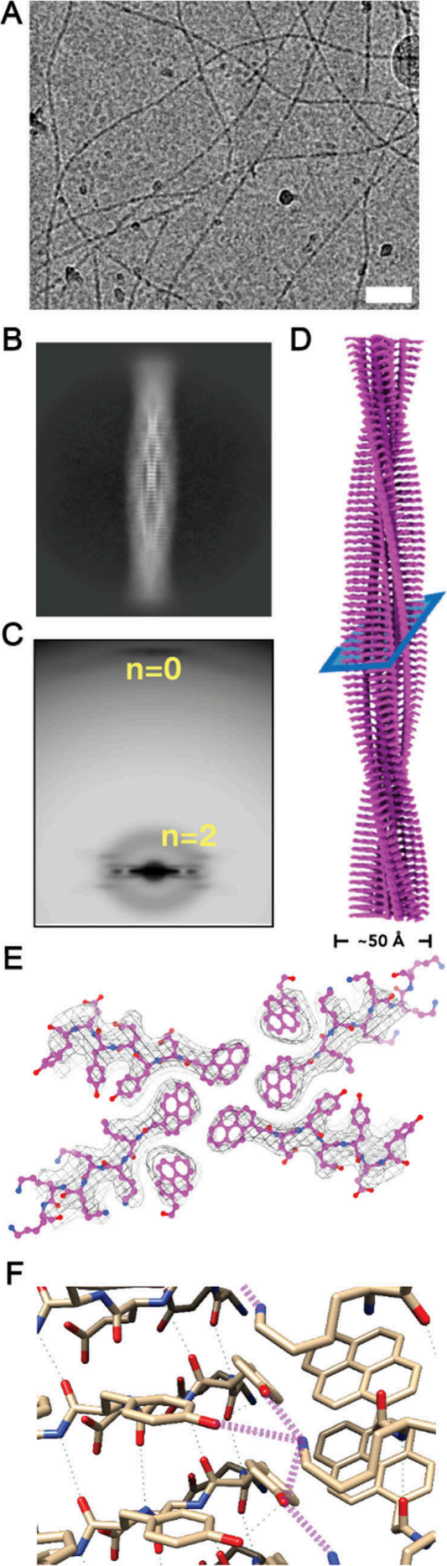
(A) Cryo-EM image of peptide nanofiber at pH 10. (Scale bar = 50 nm). (B) 2D average of the nanofiber. (C) Averaged power spectrum from particles aligned to the 2D average. (D) 3D helical reconstruction of nanofiber. (E) Cross-section as shown in panel (D), with atomic model fit into the cryo-EM map. (F) Intermolecular hydrogen bonds between lysine and tyrosine (magenta).

Morphological changes (Figure 2C) correspond to fluorescent and CD spectra. TEM images show that Pyn-EYEYEY (**2**) forms nanoparticles, consistent with its disordered nature (Figure S6). However, significant fiber formation occurs when 0.5 equiv of Pyn-KKKKKK (**1**), which is amorphous alone (Figure S6), is mixed with Pyn-EYEYEY (**2**) (Figures 2C and S7). At an equimolar mixture, further fiber coalescence is observed, resembling a braid as the concentration of **1** reaches 2 equiv. Adding an excess of **1** to the equimolar mixture results in fiber bundling, highlighting the context-dependent interactions of intrinsically disordered peptides (Figure 2D).

Controls with 0.1–2 equiv of **1** added to 500 μM of compounds **2**, **3**, **4**, and **5** (Figure S7–S10) confirm that positively charged **1** induces fiber formation in these mixtures, with bundles forming as more **1** is added. Higher tyrosine content reduces the amount of **1** needed for abundant fiber formation; for example, compound **2**, with three tyrosines, requires only 0.1 equiv, while compound **5**, without tyrosine, needs 0.5 equiv.

The key insight into heterotypic intrinsically disordered peptide assemblies is the atomistic structure of their nanofibers. Recent advances in cryo-EM enable the resolution of peptide assemblies. structures,^30,49−51^ including amyloid fibrils.^25^ Using cryo-EM, we elucidated the nanofiber structures of the peptide mixtures. Morphological assessments across pHs (4, 7.4, and 10) showed consistent fiber diameters, though low pH favored bundle formation (Figure S20). Cryo-EM datasets at pH 7.4 and 10 revealed identical 2D class averages, confirming a single filament architecture. Improved filament separation at pH 10 allowed for detailed imaging, showing a homogeneous, single type of cross-β filament with a diameter of ∼50 Å (Figures 3A–D). Testing all possible indexed symmetries established that the nanofiber has C_2_ symmetry, with a helical rise of 4.68 Å and a twist of −4.22°, featuring parallel cross-β packing and six hydrophobic pyrene rings in the core (Figure 3E). The nanofiber’s asymmetric subunit (ASU) contains three peptide copies: one Pyn-KKKKKK (**1**), one Pyn-EYEYEY (**2**), and a third with only an ordered pyrene ring. The remaining residues of the third peptide were flexible and disordered, revealing a disorder-to-order continuum and demonstrating unexpected conformational heterogeneity in heterotypic intrinsically disordered peptide nanofibers.

Moreover, analysis of the atomic model revealed multiple hydrogen bonds between tyrosine and lysine (Figure 3F), and we have seen side-chain densities from the cryo-EM map to support those side-chain conformations. As shown in Figure S22B, K^1^ forms three intermolecular hydrogen bonds with Y^2^ and Y^4^ from the same peptide and Y^2^ from another peptide, and tyrosine also forms an intramolecular hydrogen bond between Y^2^ and Y^4^. The conformationally disordered Pyn-EYEYEY and Y^6^ from the ordered Pyn-EYEYEY should allow hydrogen bonding with additional Pyn-KKKKKK, which explains the postassembly bundle formation. To verify this, we also synthesized Pyn-EFEFEF (**6**) to interact with Pyn-KKKKKK and found that postassembly addition of Pyn-KKKKKK was unable to form well-defined bundles (Figure S11). This result indicates that intermolecular hydrogen bonding between lysine and tyrosine, as well as conformational flexibility, are crucial for the post-assembly bundling of nanofibers. Notably, Y–K interactions play a key role in the action of protein lysine methyltransferases.^52^

This work provides an atomistic understanding of heterotypic assemblies of intrinsically disordered peptides and their unique context-dependent behaviors. Our findings emphasize the critical role of aromatic side chain quantity and peptide stoichiometric ratio in determining the morphology and stability of these assemblies. The most revealing feature is the partial preservation of conformational flexibility, a disorder–order continuum, and a high number of hydrogen bonds between lysine and tyrosine within the nanofibers of intrinsically disordered peptides. While replacing pyrene with other hydrophobic aromatic residues may achieve similar assemblies, multiple residues are required for sufficient interactions. The conformational dynamics are sequence-dependent and anisotropic in the fibers. That is, the disordered portion no longer uniformly distributes on the radial surfaces of the fibers. Although the structure of the bundles formed by post-assembly addition of **1** is beyond current cryo-EM capabilities, the revealed filament’s partial disorder likely enables further interfilament interactions mediated by **1** to form bundles. These insights gained from this research enhance our comprehension of IDR interactions and offer a versatile approach to dissecting the complex behaviors of disordered protein regions with potential applications in materials science and cellular function modulation.

## Supporting Information Available

Detailed experimental procedures and materials; HPLC and MS spectra for individual compounds; fluorescence emission spectra and CMC fitting results for each compound; TEM images, CD spectra, and fluorescence spectra for additional peptide mixtures; cryo-EM images with power spectrum, 2D averages, and refinement statistics for the atomic model (PDF)
