# Supplementary material for: Context-Dependent Heterotypic Assemblies of Intrinsically Disordered Peptides
Source: J Am Chem Soc. 2025 Jan 14;147(4):2978–83. doi: 10.1021/jacs.4c12150 (PMC11841035; doi:10.1021/jacs.4c12150)
Supplement: Supplementary file 1 — ja4c12150_si_001.pdf [file ja4c12150_si_001.pdf]

Supplemental Information

Context-Dependent Heterotypic Assemblies of Intrinsically Disordered Peptides

Yuchen Qiao[1], Ayisha Zia[2], Grace Wu[1], Zhiyu Liu[1], Jiaqi Guo[1], Matthew Chu[1], Hongjian He[1], Fengbin Wang[2],* Bing Xu[1]*

[1]Department of Chemistry, Brandeis University, 415 South St., Waltham, MA 02454, USA

[2]Department of Biochemistry and Molecular Genetics, University of Alabama at Birmingham, Birmingham, AL 35233, USA

Table of Contents

Supplemental Experimental Procedures 3

Supporting data 6

Supplemental Experimental Procedures

Materials

2-Cl-trityl chloride resin (1.02 mmol/g) and Fmoc protected amino acid building blocks were purchased from GL Biochem (Shanghai, China).O-benzotriazole-N,N,N,N0-tetramethyluronium-hexafluorophosphate (HBTU) was purchased from Chem impex. N, N-diisopropylethylamine (DIPEA) were purchased from TCI America. 1-pyreneacetic acid was purchased from Sigma-Aldrich. Dimethylformamide (DMF), methylene chloride (DCM), trifluoroacetic acid (TFA), methanol (MeOH), triethylamine, and other reagents and solvents were purchased from Fisher Chemical. All reagents and solvents were used without further purification.

Instruments

All crude compounds were purified using a reverse phase HPLC (Agilent 1100 Series) with HPLC grade water (0.1% TFA) and HPLC grade acetonitrile (0.1% TFA) as eluents. LC-MS spectra were obtained on a Bruker timsTOF Pro Mass Spectrometer equipped with an Elute UHPLC chromatograph. TEM was conducted on Morgagni 268 transmission electron microscope. CD was conducted on Jasco J-810 spectropolarimeter. Fluorescence emission spectra were obtained using Shimadzu RF-5301PC spectrometer.

Peptide synthesis

Compound **1-6** were synthesized via solid-phase peptide synthesis (SPPS) (Scheme S1). 2-Cl-trityl chloride resin was dipped in methylene chloride (DCM) for 5 min, followed by loading the amino acid building blocks using N, N-diisopropylethylamine (DIPEA) in DCM overnight. A capping solution (DCM:MeOH:DIPEA = 17:2:1) was added for 30 min, and 20% piperidine in dimethylformamide (DMF) was added for another 30 min for deprotection. For subsequent couplings, amino acids, HBTU, HOBt, and DIPEA were loaded for 2 h, with washing steps with DMF after each coupling. Peptides were cleaved using trifluoroacetic acid (TFA) and left to react for 1 h. After concentrating the reaction mixtures, ethyl ether was added for peptide precipitation. The crude peptides were then purified by HPLC. Peptides **1-6** were produced in multiple milligram scales with yields of 90%, 73%, 65%, 61%, 95%, and 78%, respectively.

Fluorescent spectra measurement

The fluorescence emission spectra of 400 µL solutions for each sample were recorded from 360 nm to 600 nm. All fluorescence emission spectra were obtained using Shimadzu RF-5301PC spectrometer with an excitation wavelength of 355 nm.

Critical micelle concentration (CMC) measurement

The critical micellar concentrations (CMC) were assessed utilizing the fluorescent spectra of pyrene motif on the peptides. Various concentrations of a peptide were prepared in distilled water. By plotting the intensity ratios at 374 nm and 384 nm (I_1/I_3) for each concentration, the concentration at the intersection point of two fitted lines was identified as the CMC.

E/M analysis

The fluorescence spectra of each peptide were divided into a monomer peak (M) from 360–430 nm and an excimer peak (E) from 430–600 nm for integration using the "Area Under Curve"

analysis feature in GraphPad Prism 9. The resulting E/M ratio was then plotted using the same software.

Transmission electron microscopy (TEM) sample preparation
Negative stain electron microscopy for most of the study due to its speed, convenience, and lower cost compared to cryo-EM. After placing 5 μL samples on 400 mesh copper grids coated with continuous thick carbon film (~35 nm) which was glowed discharged, we washed the grid with ddH_2O and UA (uranyl acetate). The sample loaded grid was stained with the UA for 20 seconds. The residual UA was removed by filter paper and then dried in air. TEM images were obtained with FEI Morgagni 268 80 kV with a 1 k × 1 k AMT CCD camera.

Circular Dichroism (CD)
CD spectra were recorded (180–300 nm) using a JASCO 8-10 spectrometer under nitrogen atmosphere. Peptide **1-5** and/or mixture solution were added into a quartz cuvette (1 mm) and scanned with 1 nm interval at the scanning speed of 100 nm/min. Each measurement was accumulated twice, with the Savitzky-Golay smoothing method applied using a convolution width of 13.

Sample preparation
Peptides were dissolved in double-distilled water at 10 mM as stock solution, with the pH adjusted to 7.0 using 1 M hydrochloric acid or sodium hydroxide. The stock solution was diluted to the desired concentration with double-distilled water for reactions. Mixtures were prepared by combining equal volumes of two components at the desired concentrations (e.g., "500 μM Pyn-EYEYEY + 2 equiv. Pyn-KKKKKK" was made by mixing equal volumes of 1 mM Pyn-EYEYEY and 2 mM Pyn-KKKKKK). Context-dependent mixtures (Figure 2D) were performed by adding stock solution to the aforementioned mixture system. All the samples were prepared and incubated at room temperature before characterization.

Cryo-EM and image processing
The peptide sample was vitrified on glow-discharged lacey carbon grids using a GP2 plunge freezer (Leica). Data were collected on a Titan Krios (Thermo Fisher) equipped with a K3 camera (Gatan). 16,245 micrographs were collected using 1.07 Å per pixel The imagine session were done under the electron-counting mode, using a defocus range of 1-2 μm with ~50 electrons/Å^2 distributed into 40 fractions. Motion correction[1] and contrast transfer function (CTF) estimation[2] were performed within cryoSPARC[3]. Particles were auto-picked using the "filament tracer" function with an 11-pixel shift. Iterative rounds of 2D classification were used to remove junk particles, such as carbon edges and averages without high resolution features. A list of possible helical symmetries was calculated from the averaged power spectra of raw particles, and tested systematically by trial-and-error until recognizable peptide features were observed[4]. Resolution estimation was carried out using map:map Fourier Shell Correlation (FSC), model:map FSC, and d99 criteria[5] on B-factor sharpened maps. EMReady sharpened maps[6] were used for visualization and model building. Detailed statistics are provided in Table S1.

Model building
The filament model reached a resolution of ~ 3.3 Å, as estimated by map:map FSC. Unlike structures containing α-helices, the handedness of the cryo-EM map for a β-sheet-only structure

cannot be determined directly from the map[4]. Among reported cross-β structures made of L-amino acids, parallel β-sheets typically exhibit a left-handed twist. However, this observation may not be always true for short peptides incorporating non-standard residues. Therefore, to determine the handedness, model building was performed in both hands of the map, and the final hand was chosen based on comparation of the geometry statistics of both models, including class scores, real space correlation coefficient (RSCC), typical β-sheet hydrogen bonds, etc. The restraints for non-standard residues were obtained using eLBOW[7]. After that, peptide molecules were first manually built and adjusted in Coot[8], followed by real-space refinement in PHENIX[9]. The model fits better in the left-handed than the right-handed map, with better real space correlation coefficient (RSCC), clashscore (0.80, 34.4 vs. 0.78, 56.17, respectively) and better hydrogen bonds within β sheets. Therefore, we suggested that map probably have a left-handed twist. The resolution and refinement statistics are shown in Table S1.

Supporting data

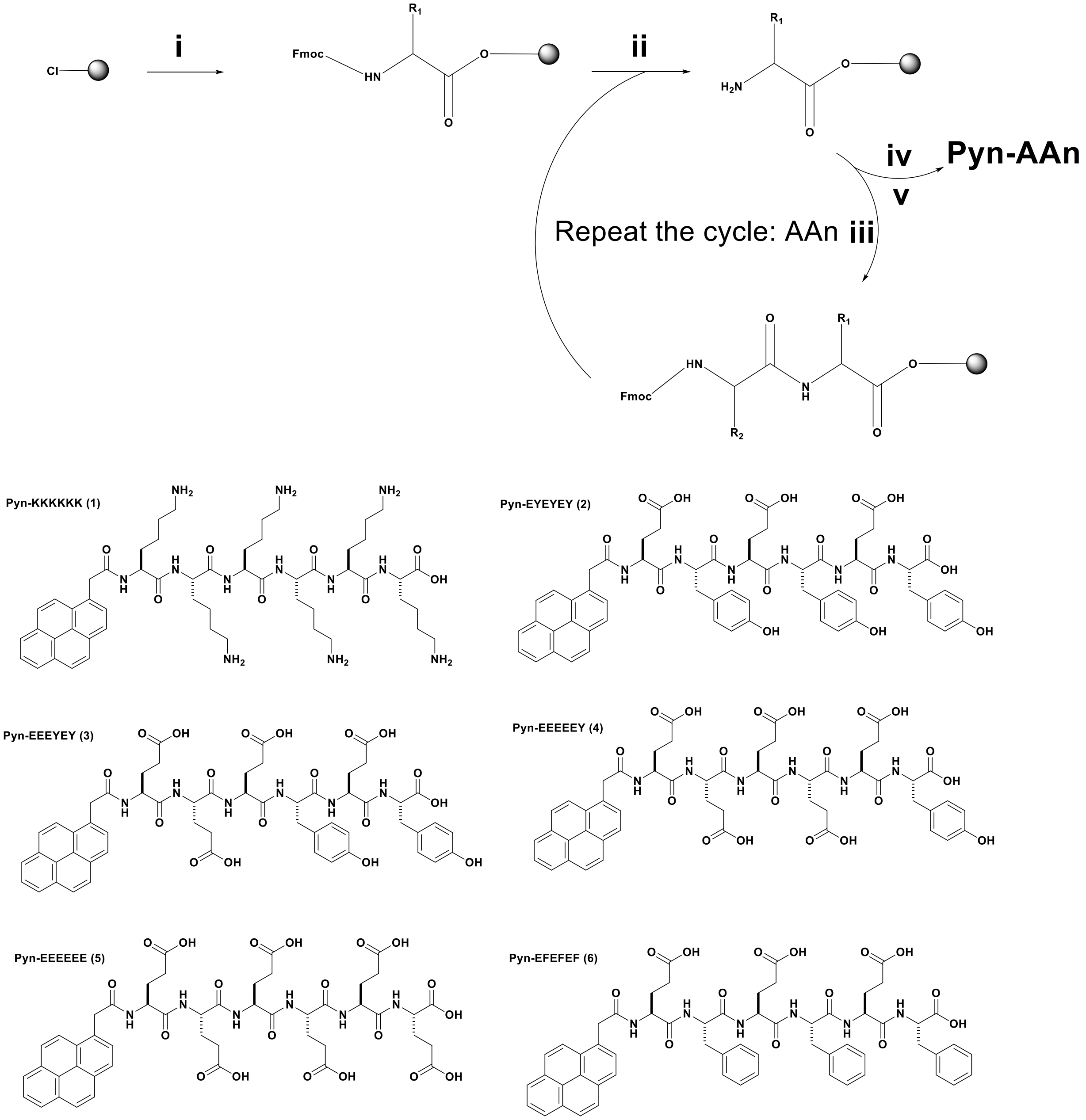

Scheme S1. Synthetic route and chemical structures for **1-6**. i) Fmoc-AA1-OH, DIPEA; ii) 20% piperidine; iii) Fmoc-AA2-OH, HBTU, HOBt, DIPEA; iv) 1-Pyreneacetic acid, HBTU, DIPEA; v) 90% TFA in water.

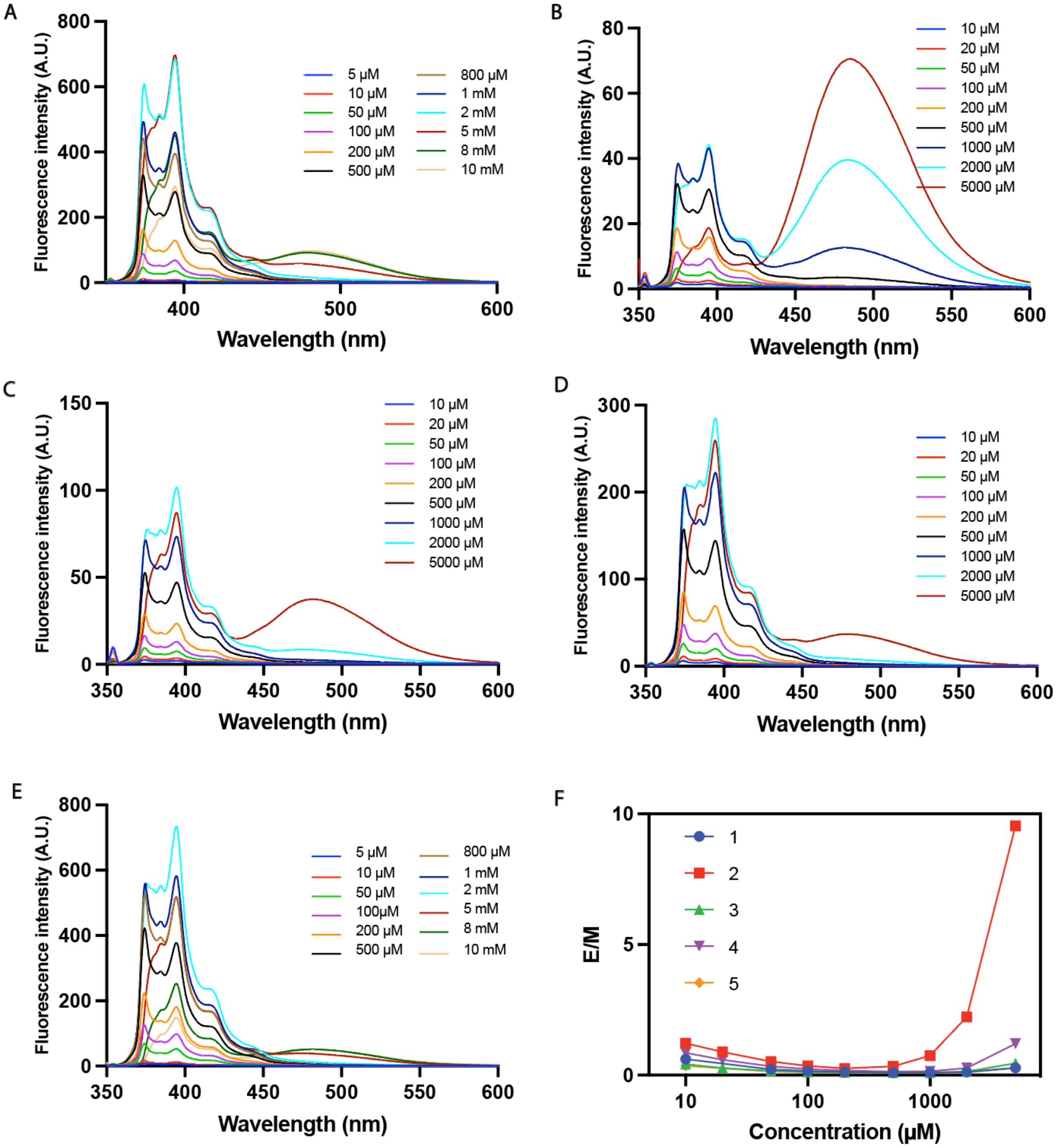

Figure S2. Fluorescent spectra of (A) **1**, (B) **2**, (C) **3**, (D) **4**, (E) **5**, and (F) Excimer/monomer (E/M) value of **1-5** at 10 μM to 5 mM in water at pH=7.

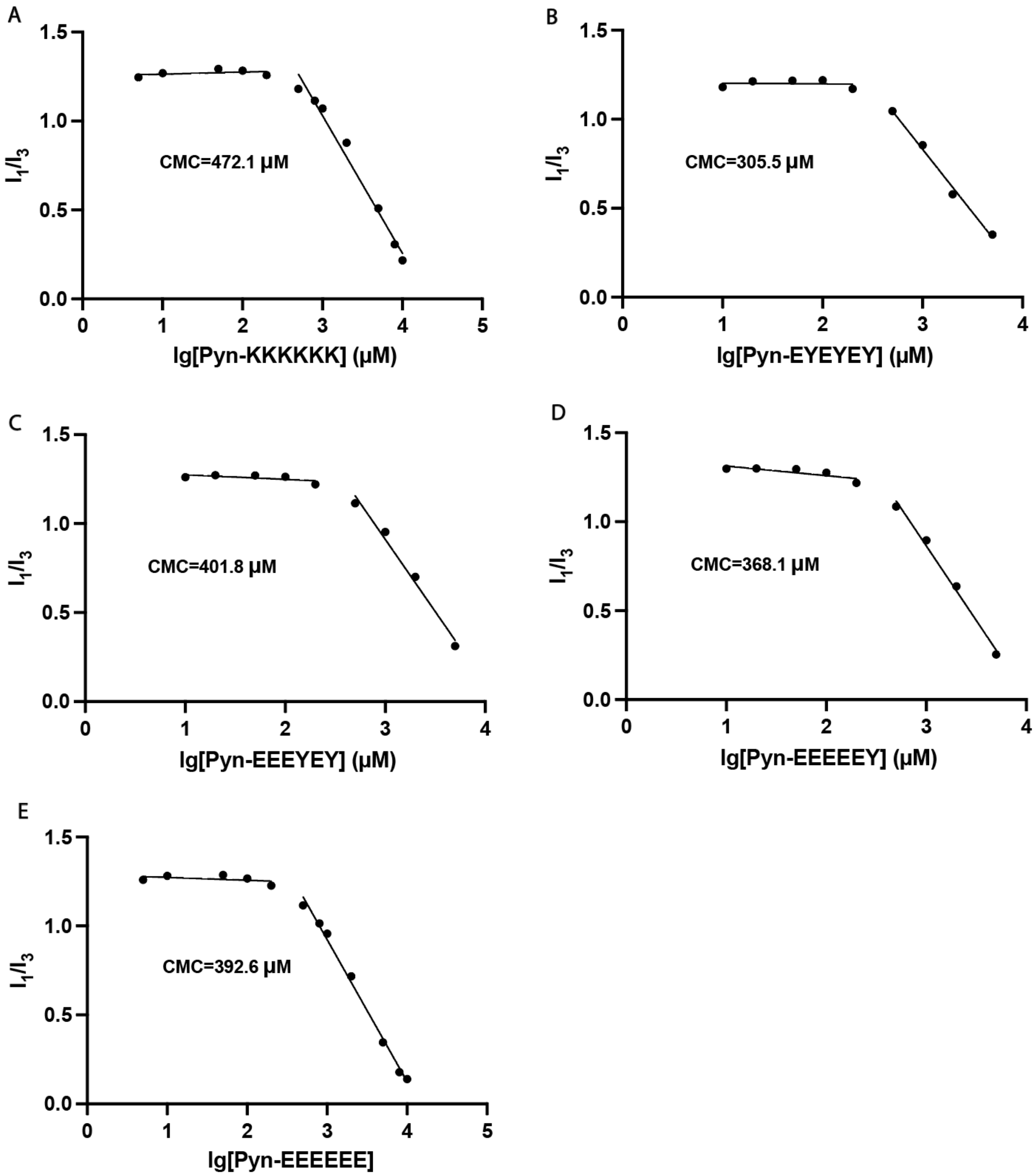

Figure S3. The fluorescence intensity ratios of (A) **1**, (B) **2**, (C) **3**, (D) **4**, (E) **5** in H_2O at 374 nm (I_1) and 384 nm (I_3).The turning points indicate the critical micelle concentrations (CMCs)

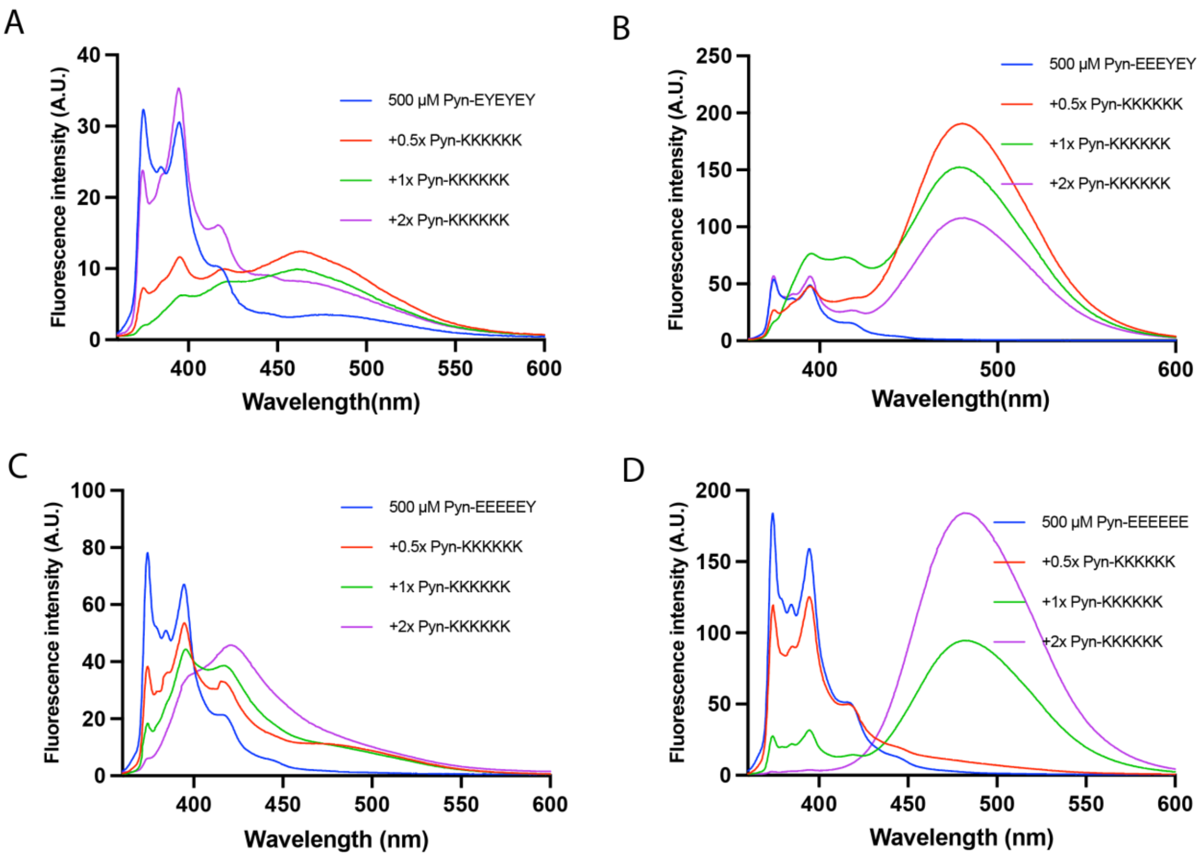

Figure S4. The fluorescent spectra of (A) **2**, (B) **3**, (C) **4**, and (D) **5** at 500 µM (blue lines) and their mixtures with **1** at the equivalence of 0.5 (red lines), 1 (green lines), and 2 (purple lines).

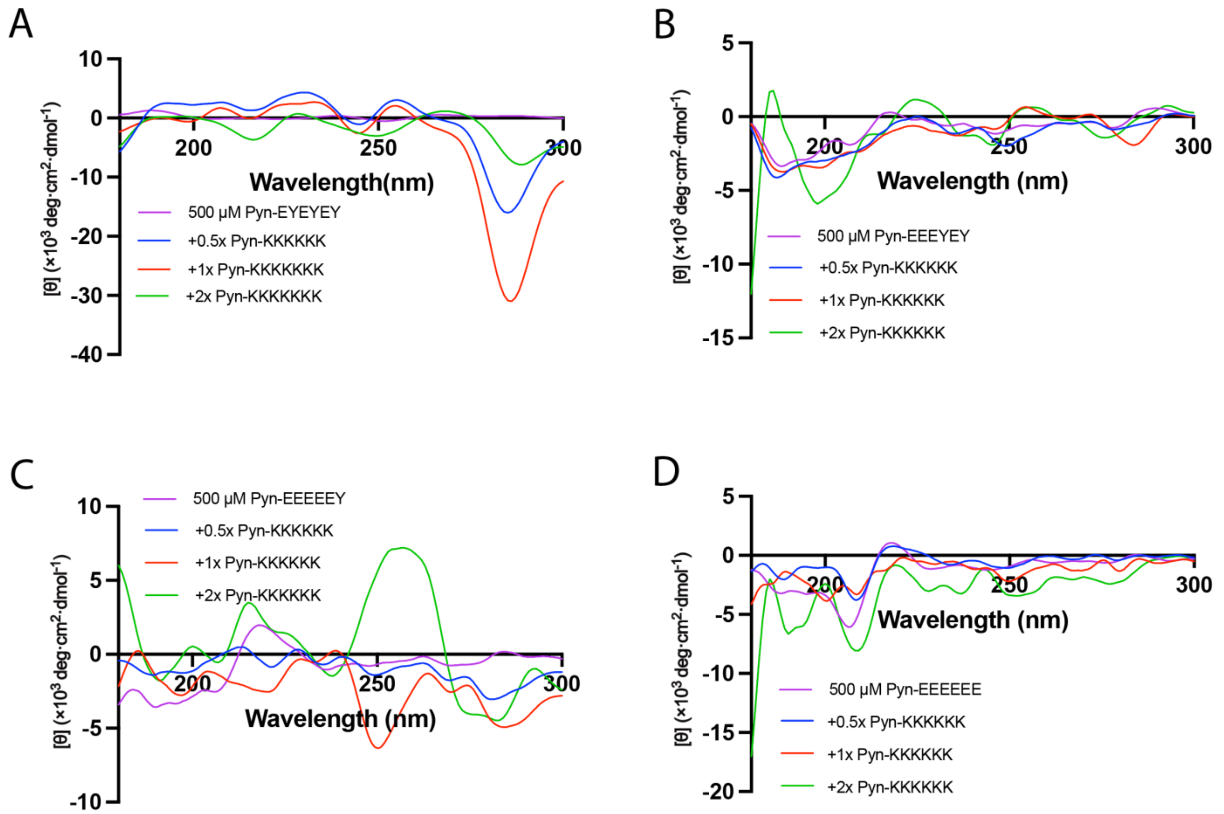

Figure S5. The circular dichroism (CD) spectra of (A) **2**, (B) **3**, (C) **4**, and (D) **5** at 500 μM (purple lines) and their mixtures with **1** at the equivalence of 0.5 (blue lines), 1 (red lines), and 2 (green lines).

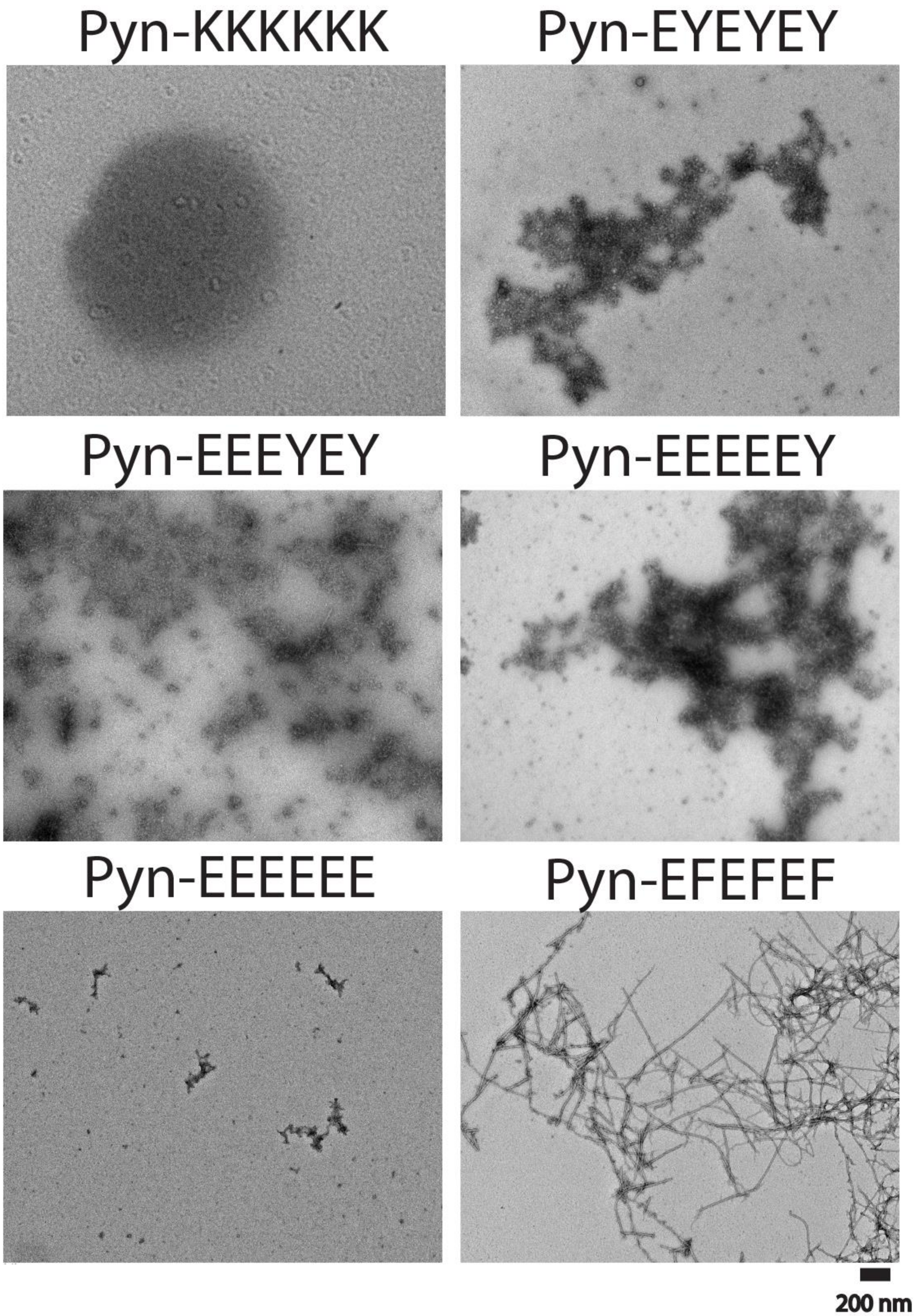

Figure S6. TEM images of IDP **1-6** at 500 μM in water.

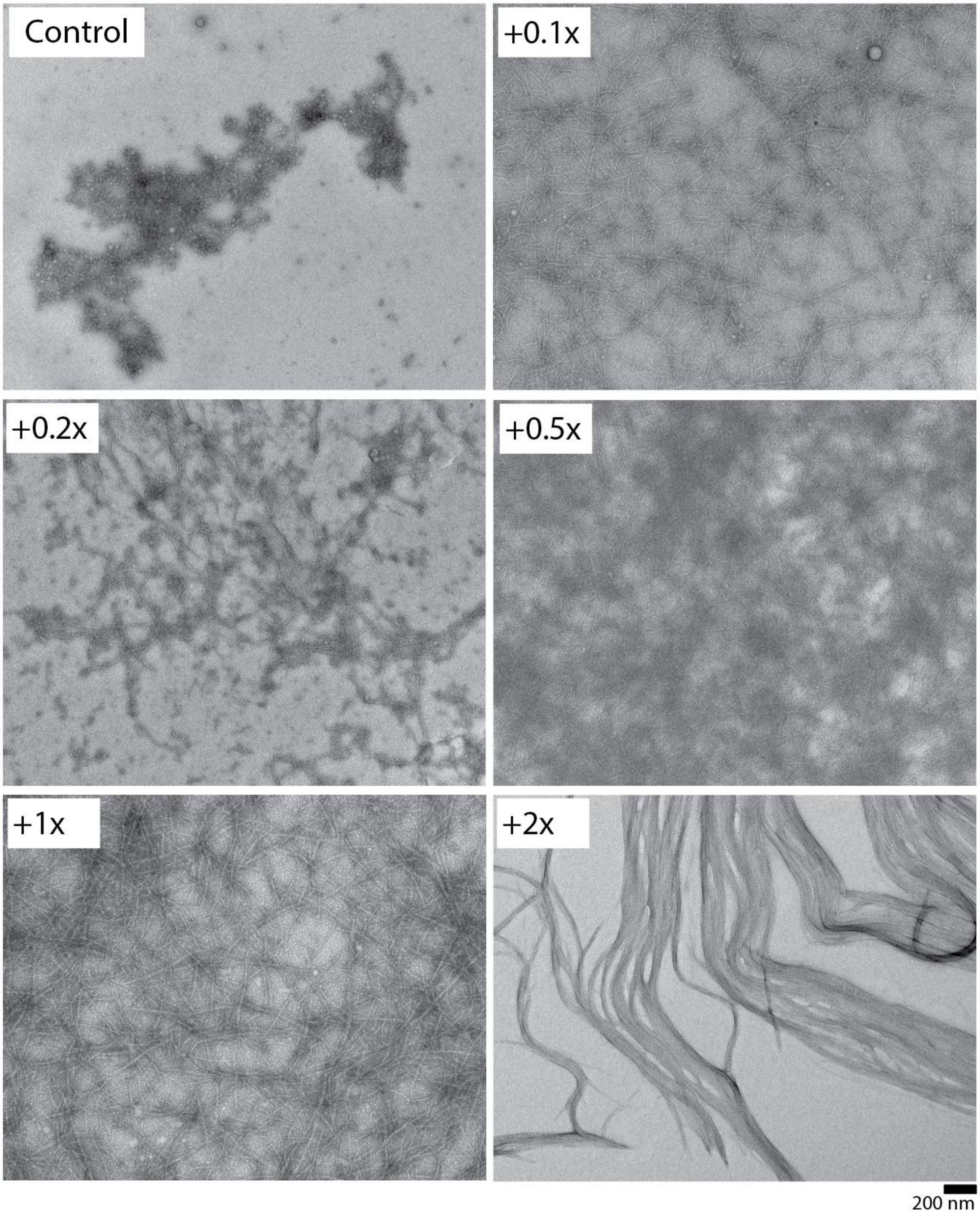

Figure S7. TEM images of **2** at 500 µM and its mixture with 0.1, 0.2, 0.5, 1, and 2 equivalence of **1** in water at pH=7 for 24 hours.

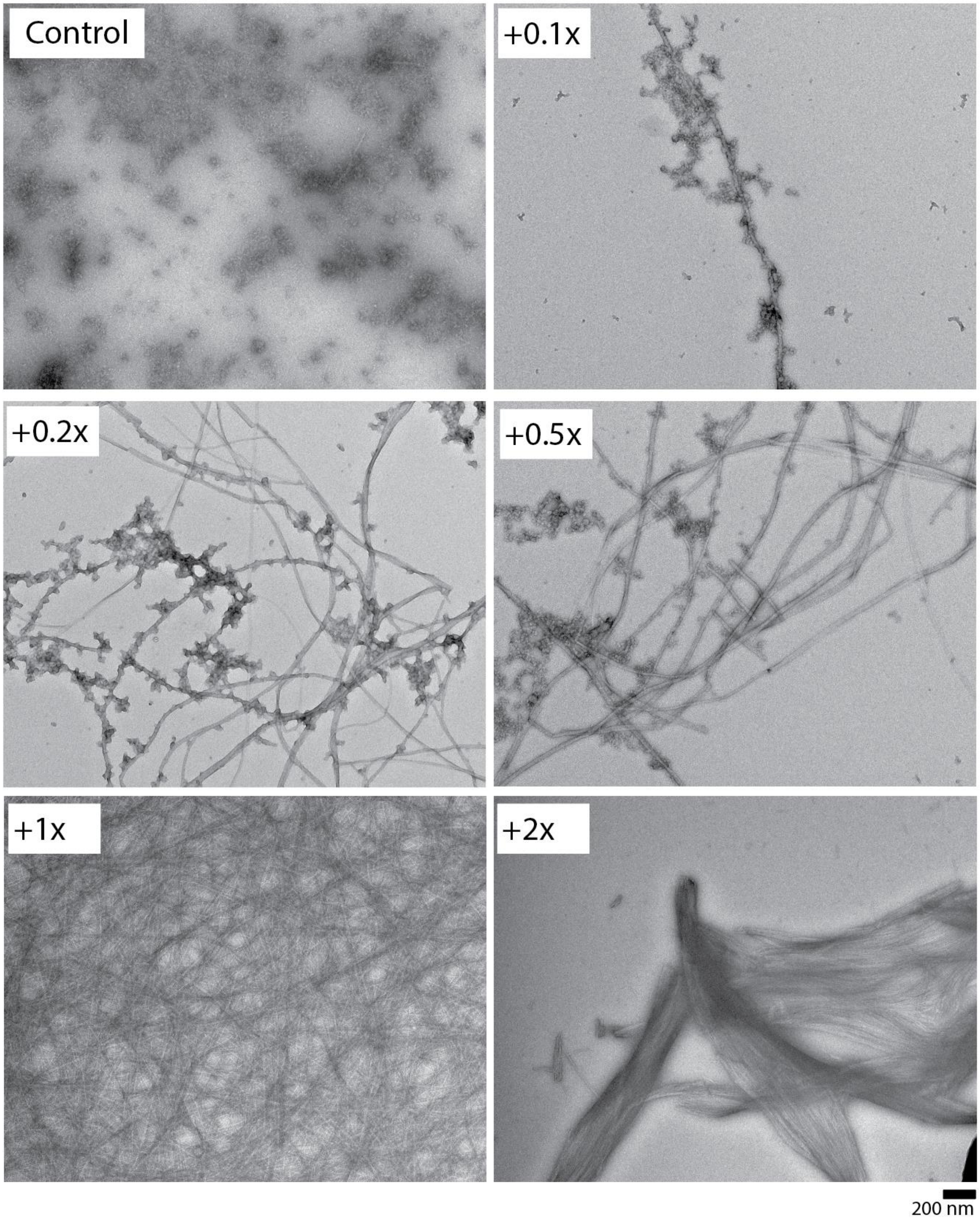

Figure S8. TEM images of **3** at 500 µM and its mixture with 0.1, 0.2, 0.5, 1, and 2 equivalence of **1** in water at pH=7 for 24 hours.

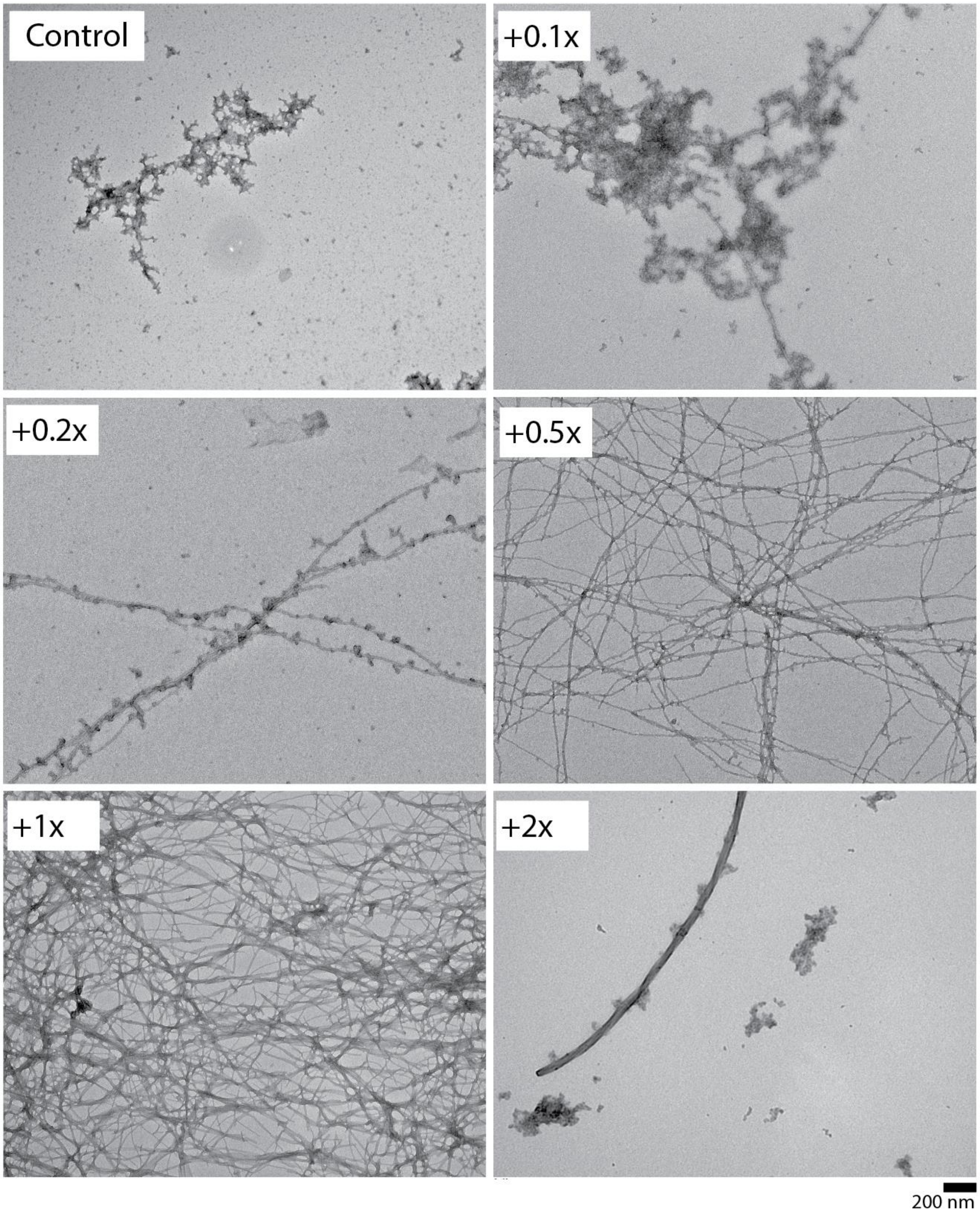

Figure S9. TEM images of **4** at 500 µM and its mixture with 0.1, 0.2, 0.5, 1, and 2 equivalence of **1** in water at pH=7 for 24 hours.

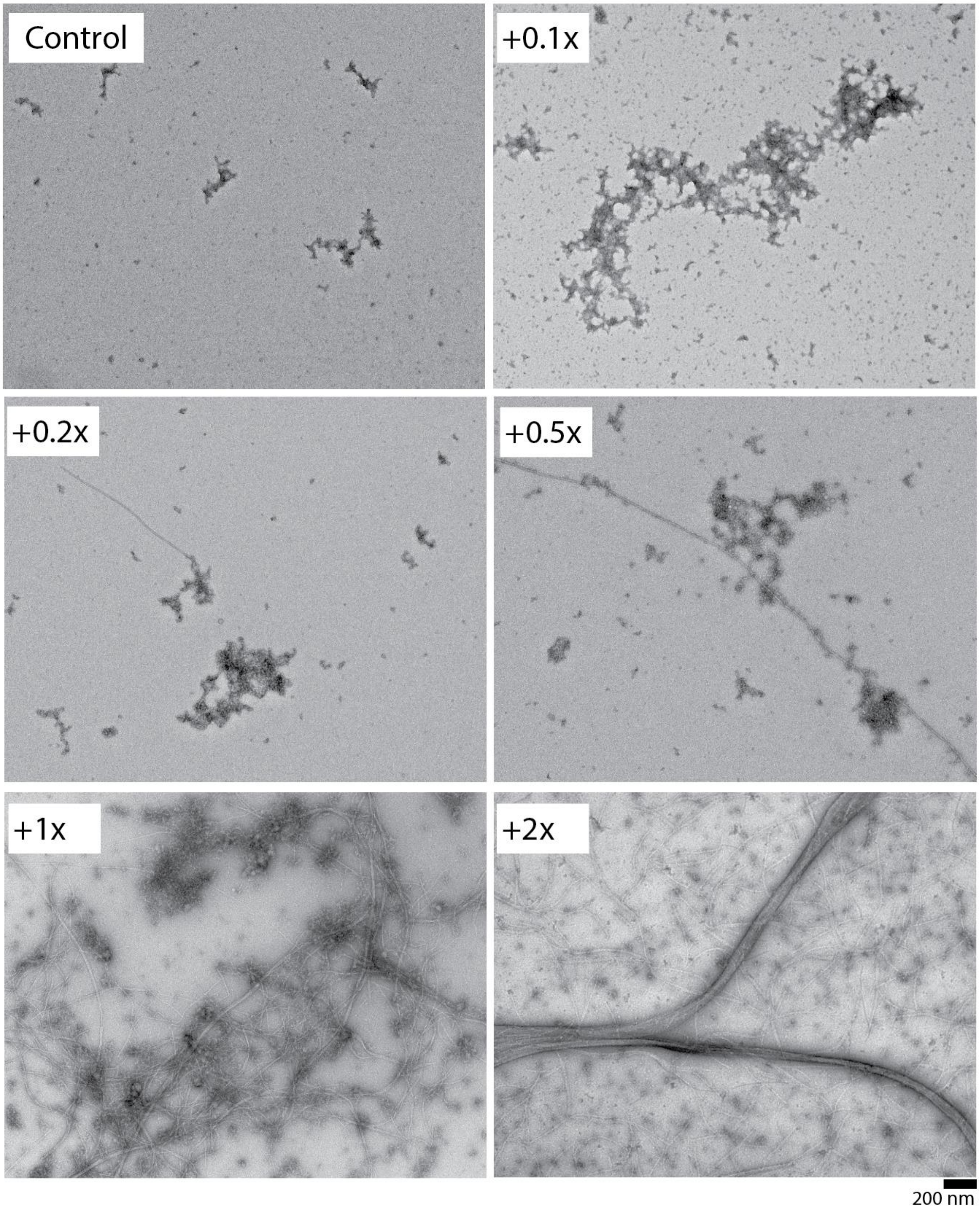

Figure S10. TEM images of **5** at 500 µM and its mixture with 0.1, 0.2, 0.5, 1, and 2 equivalence of **1** in water at pH=7 for 24 hours.

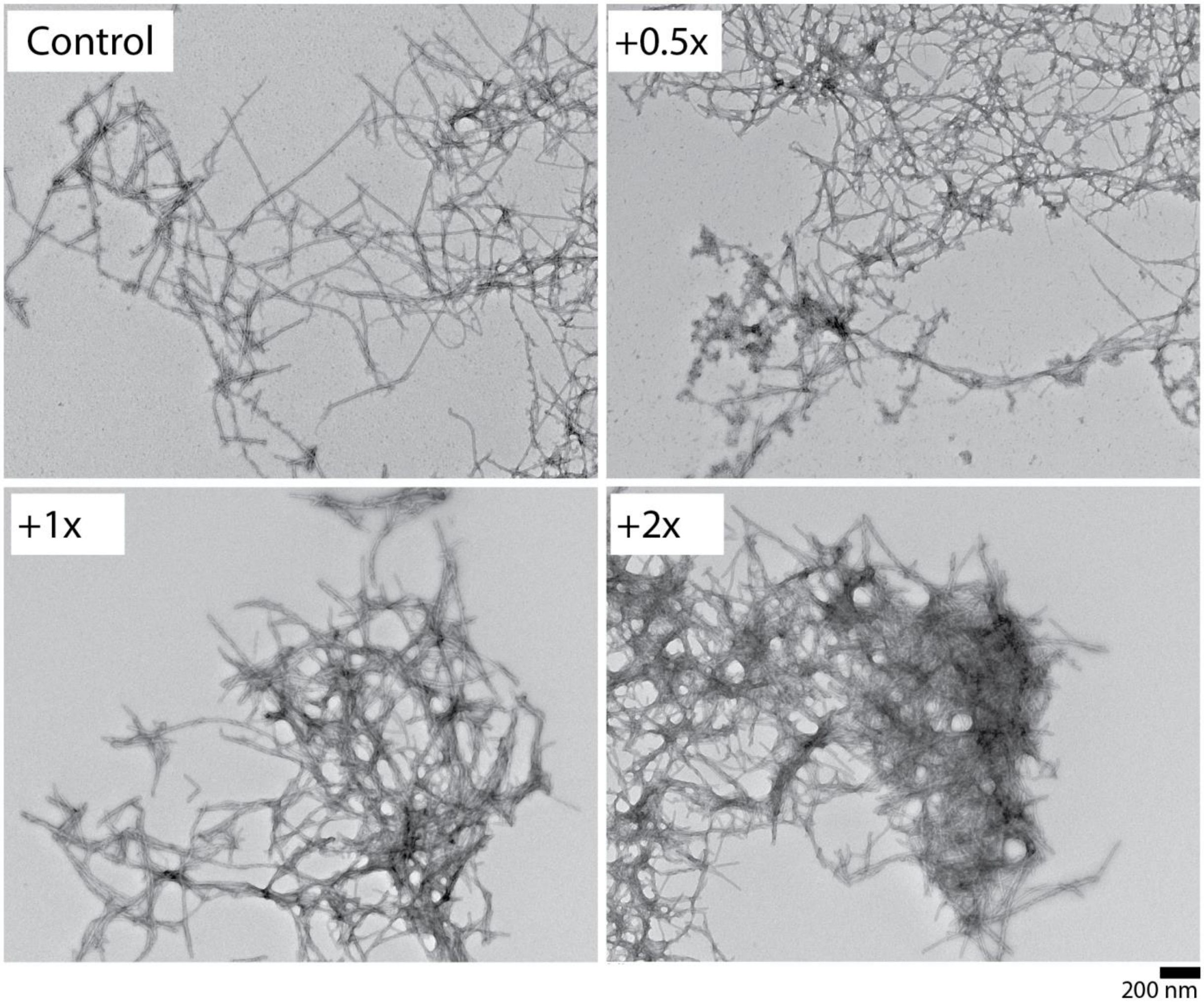

Figure S11. TEM images of **6** at 500 µM and its mixture with 0.1, 0.2, 0.5, 1, and 2 equivalence of **1** in water at pH=7 for 24 hours.

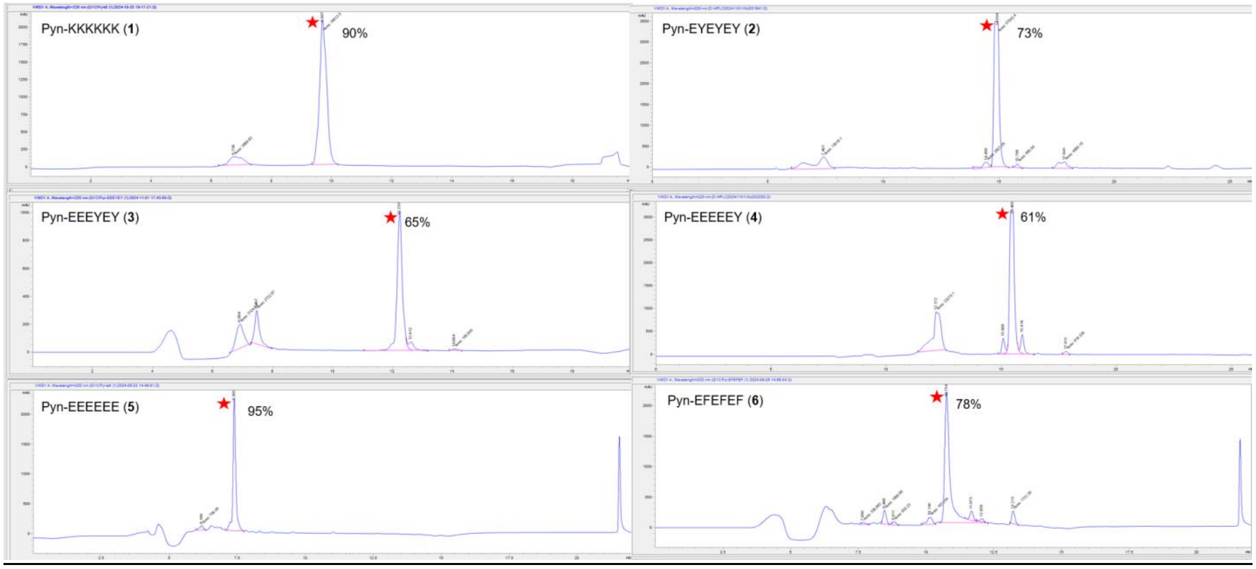

Figure S12. HPLC results for compounds 1-6. Peaks labeled with red stars correspond to the pure compounds collected, and the yields from each crude compound are noted accordingly.

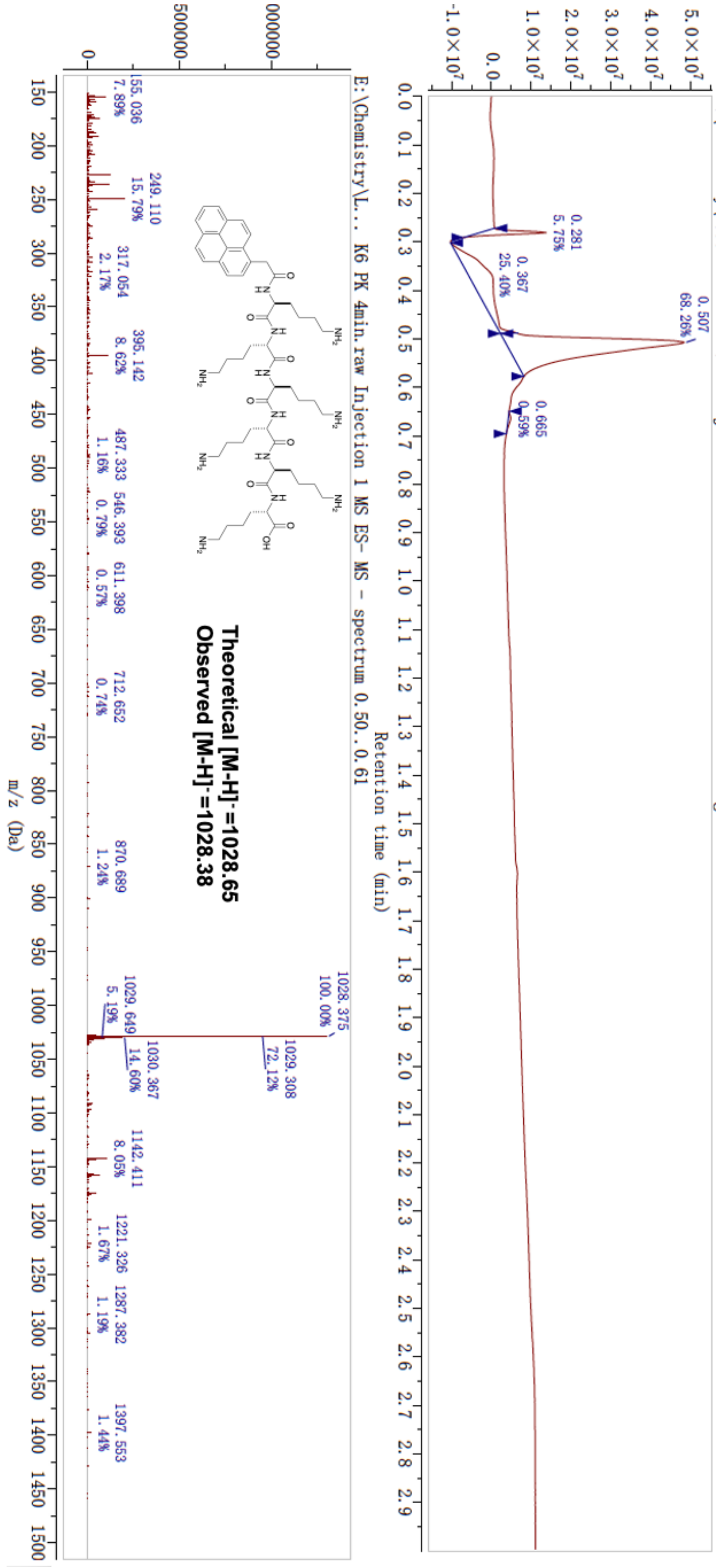

Figure S13. LC (up) and MS (down) result of **1**.

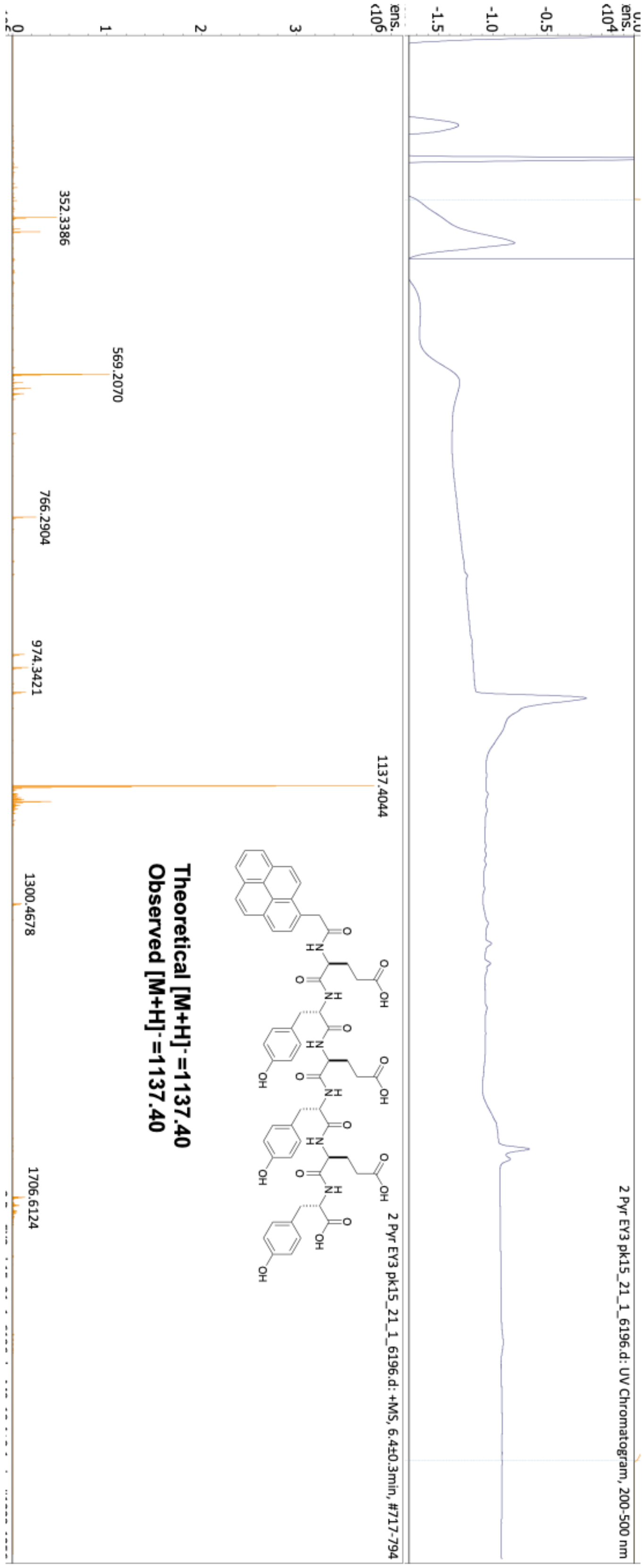

Figure S14. LC (up) and MS (down) result of **2**.

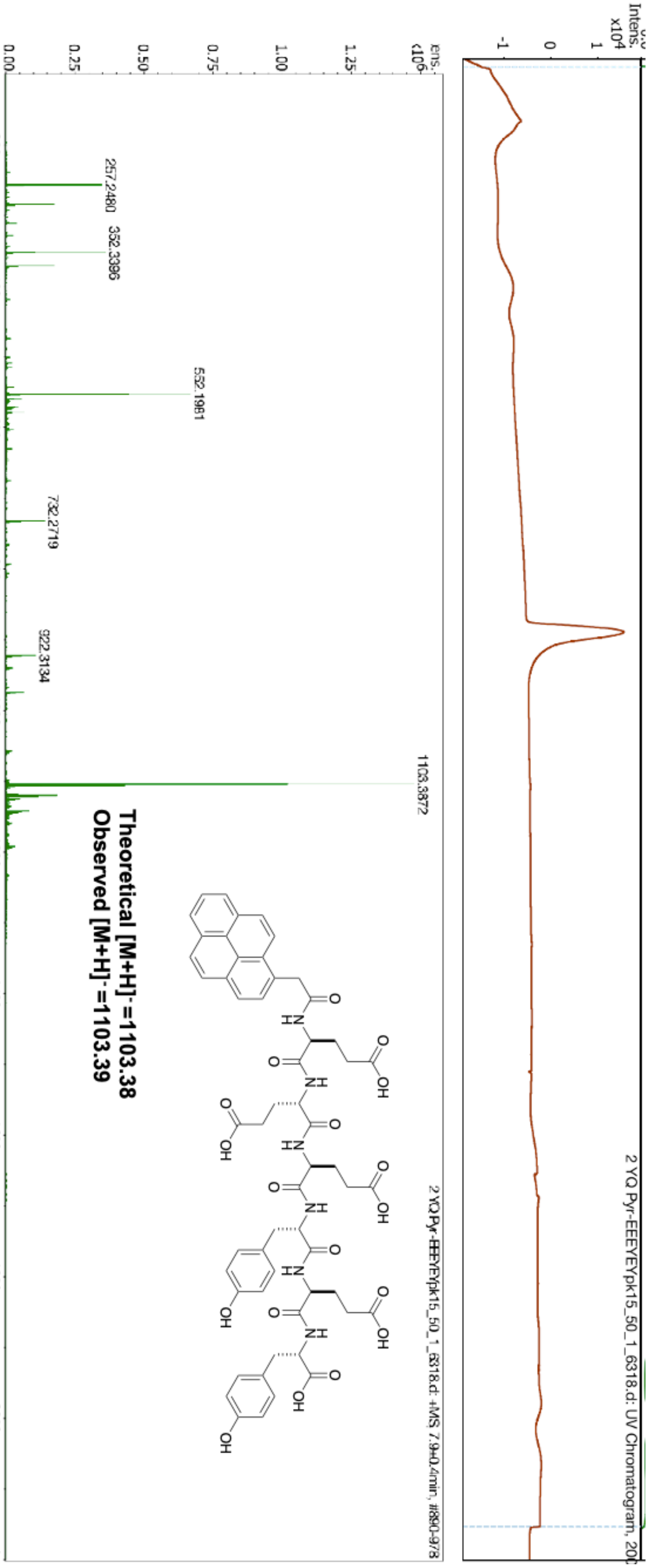

Figure S15. LC (up) and MS (down) result of **3**.

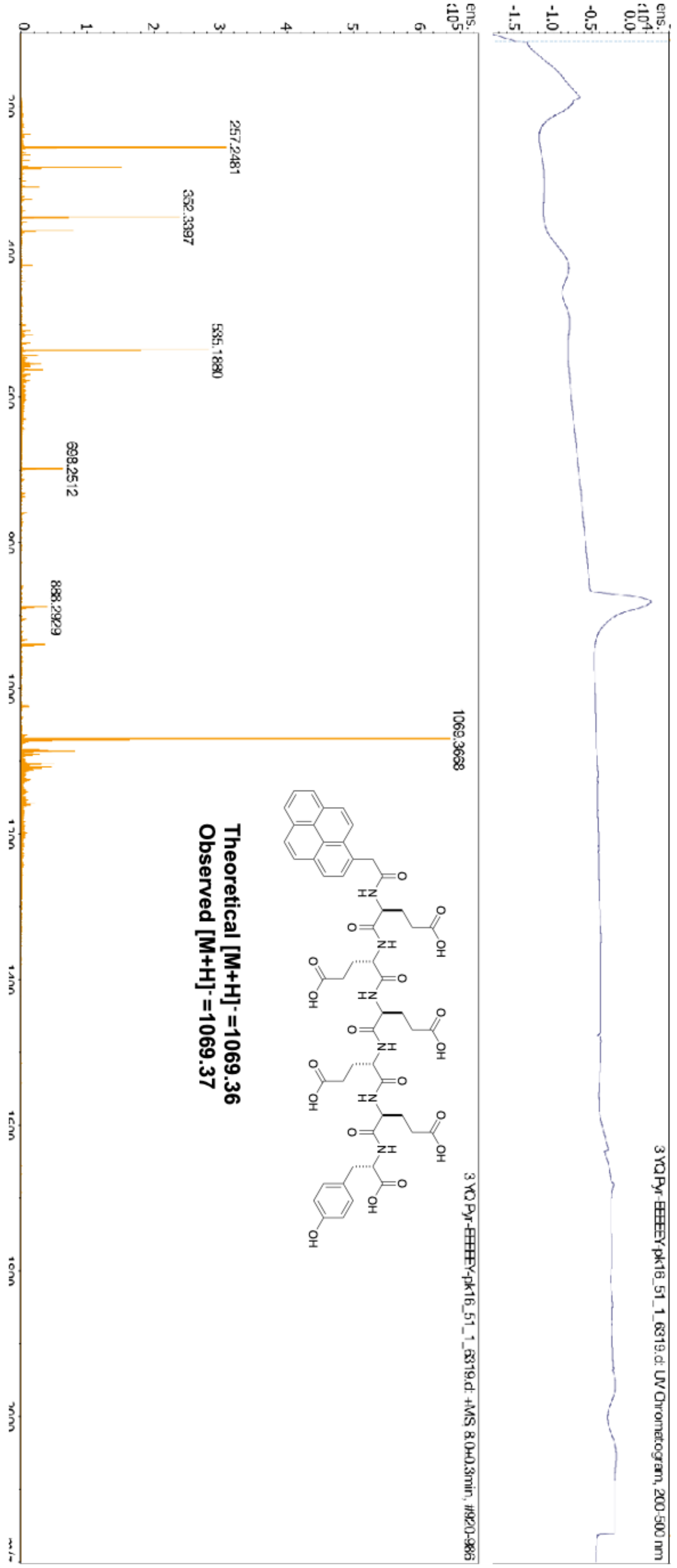

Figure S16. LC (up) and MS (down) result of **4**.

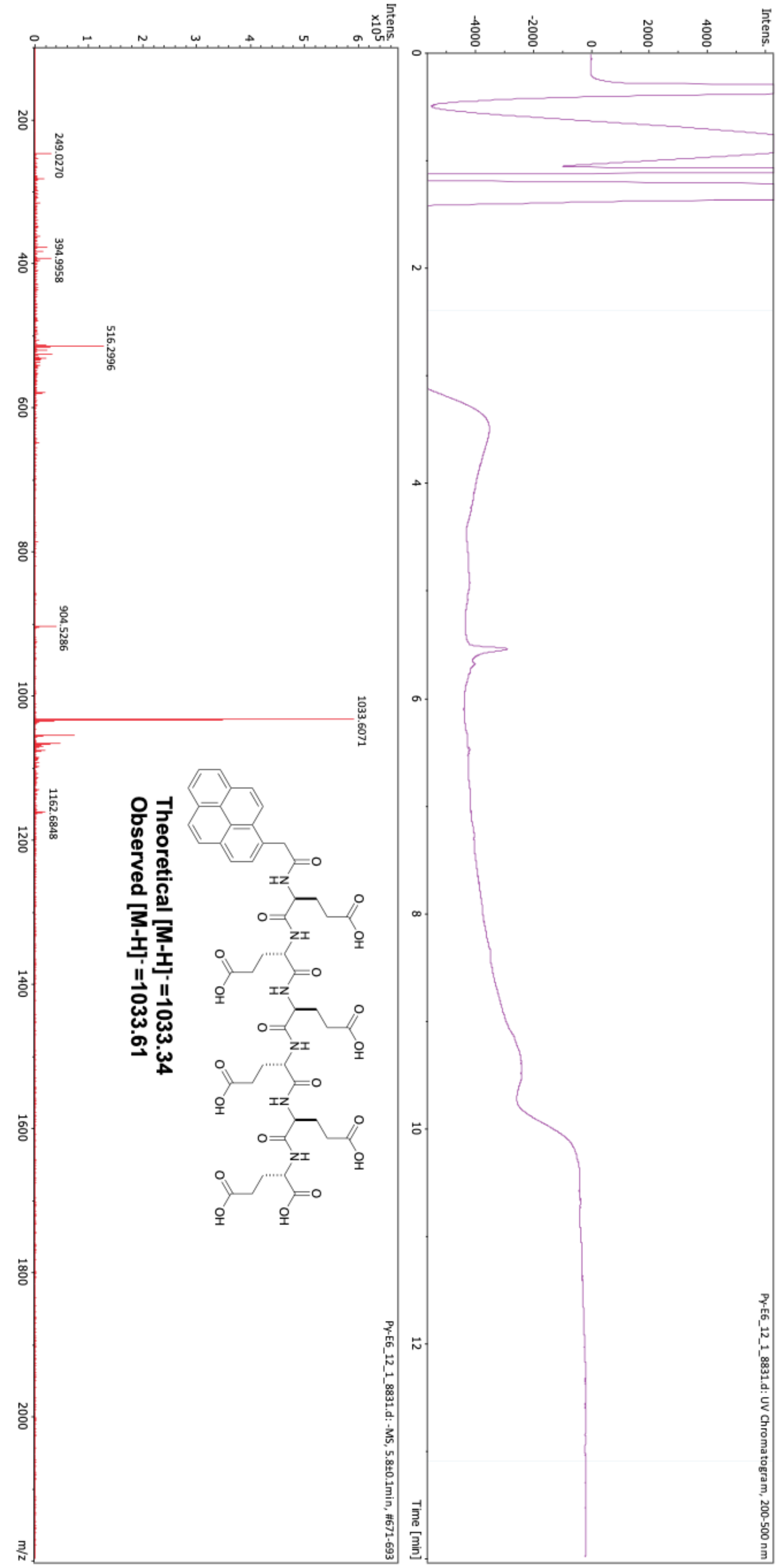

Figure S17. LC (up) and MS (down) result of **5**.

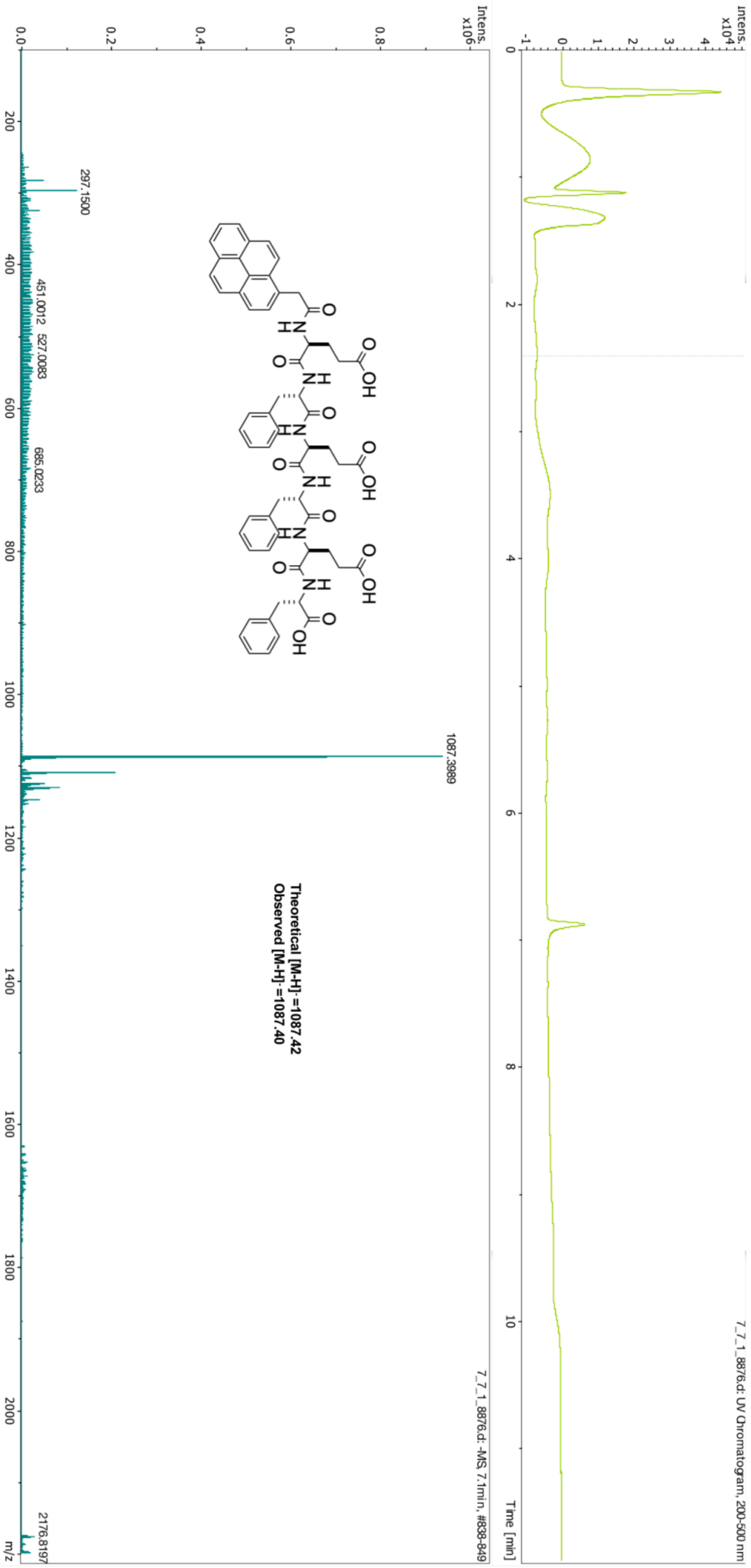

Figure S18. LC (up) and MS (down) result of **6**.

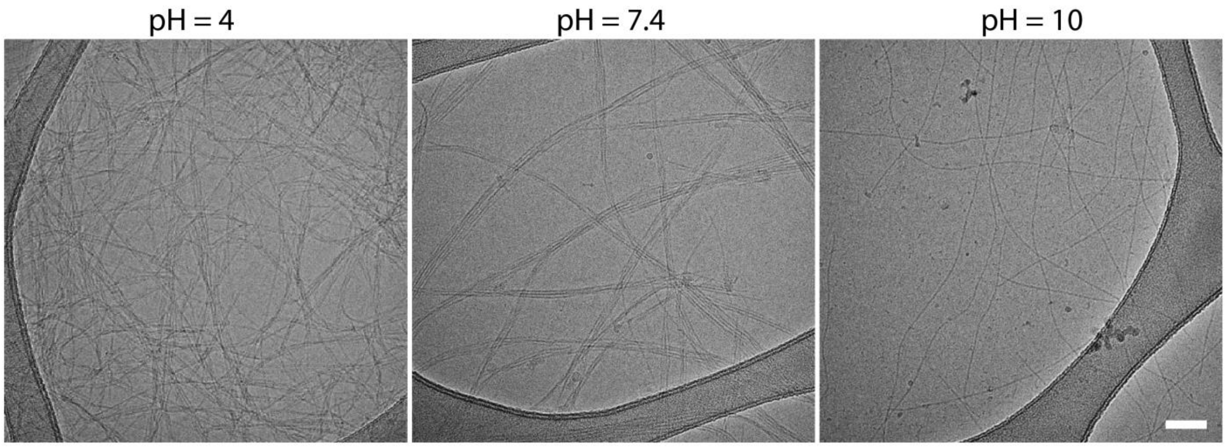

Figure S19. Cryo-EM images of equal molar mixture of **1** and **2** under pH = 4, 7.4, and 10. (Scale bar = 50 nm)

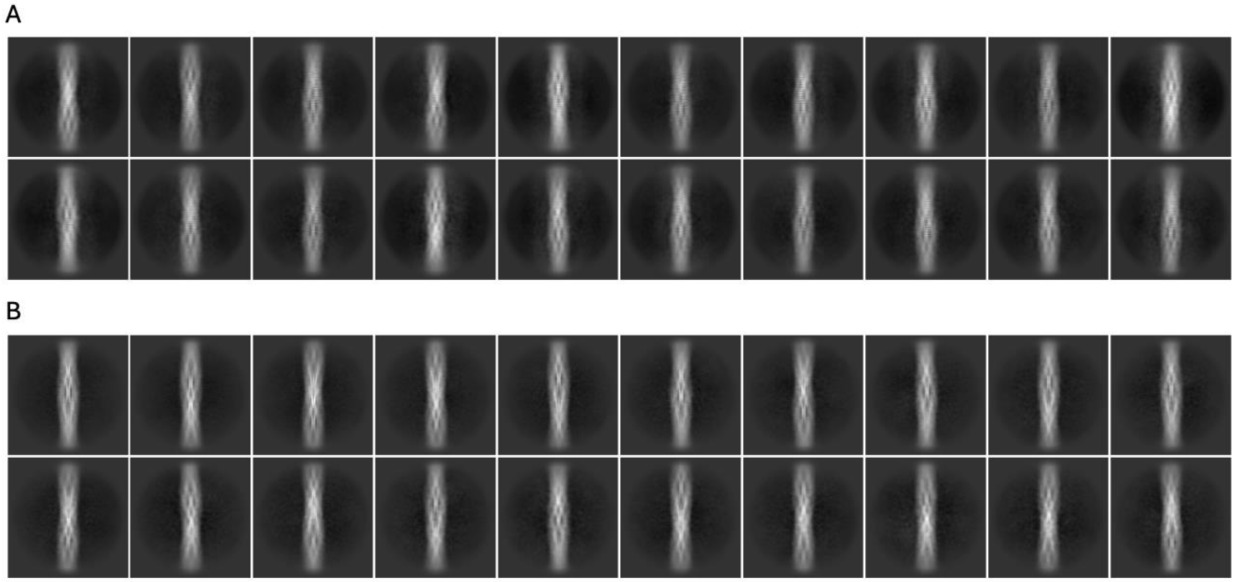

Figure S20. 2D averages of equal molar mixture of **1** and **2** under (A) pH 7.4, and (B) pH 10.

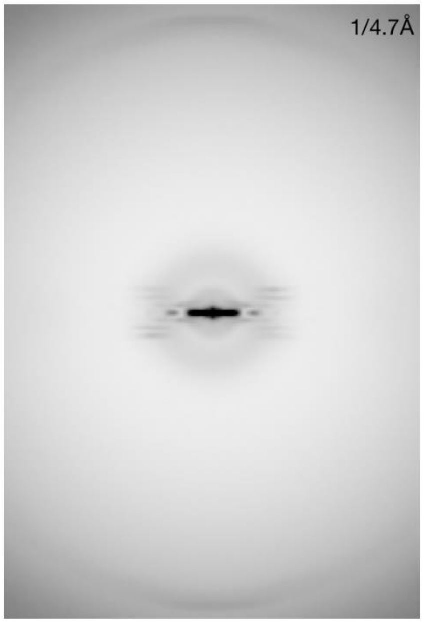

Figure S21. Power spectrum of equal molar mixture of **1** and **2** under pH 7.4.

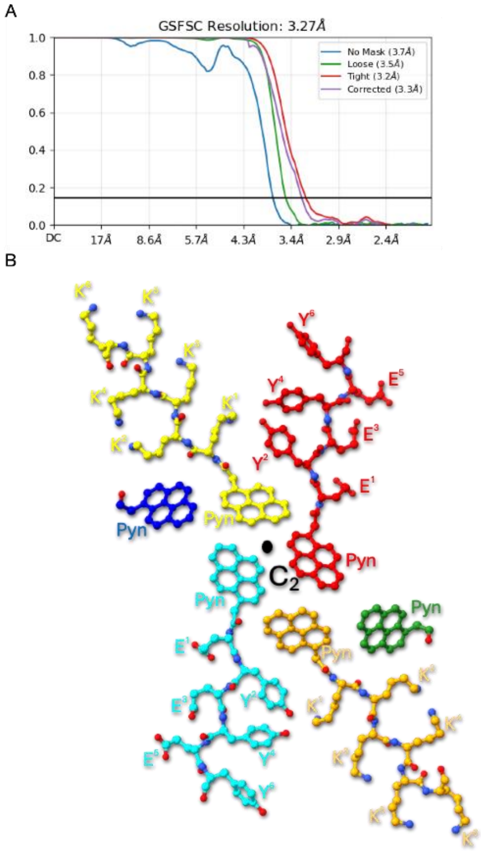

Figure S22. (A) Map:map FSC of equal molar mixture of **1** and **2** under pH 10. (B) A labelled arrangement of molecules in the nanofibers at the cross section.

Table S1. Cryo-EM and refinement statistics of equal molar mixture of **1** and **2** under pH 10.

Parameters	Pyn-KKKKKK + Pyn-EYEYEY
**Data collection and processing**	
Voltage (kV)	300
Electron exposure (e^{-} Å^{-2})	50
Pixel size (Å)	1.07
Particle images (n)	1,677,085
Shift (pixel)	11
**Helical symmetry**	
Point group	C2
Helical rise (Å)	4.68
Helical twist (°)	-4.22
**Map resolution (Å)**	
Map:map FSC (0.143)	3.3
Model:map FSC (0.5)	4.4
d_{99}	4.0
**Refinement and Model validation**	
Clashscore	34.4
Ramachandran Favored (%)	100.0
Ramachandran Outlier (%)	0.0
RSCC	0.80
**Deposition ID**	
PDB (model)	9D70
EMDB (map)	46599

References

1. Zheng, S. Q.; Palovcak, E.; Armache, J. P.; Verba, K. A.; Cheng, Y.; Agard, D. A., MotionCor2: anisotropic correction of beam-induced motion for improved cryo-electron microscopy. *Nat. Methods* **2017,** *14* (4), 331-332.
2. Rohou, A.; Grigorieff, N., CTFFIND4: Fast and accurate defocus estimation from electron micrographs. *J. Struct. Biol.* **2015,** *192* (2), 216-21.
3. Punjani, A.; Rubinstein, J. L.; Fleet, D. J.; Brubaker, M. A., cryoSPARC: algorithms for rapid unsupervised cryo-EM structure determination. *Nat. Methods* **2017,** *14* (3), 290-296.
4. Wang, F.; Gnewou, O.; Solemanifar, A.; Conticello, V. P.; Egelman, E. H., Cryo-EM of Helical Polymers. *Chem. Rev.* **2022,** *122* (17), 14055-14065.
5. Afonine, P. V.; Klaholz, B. P.; Moriarty, N. W.; Poon, B. K.; Sobolev, O. V.; Terwilliger, T. C.; Adams, P. D.; Urzhumtsev, A., New tools for the analysis and validation of cryo-EM maps and atomic models. *Acta Crystallogr., Sect. D: Struct. Biol.* **2018,** *74* (Pt 9), 814-840.
6. He, J.; Li, T.; Huang, S. Y., Improvement of cryo-EM maps by simultaneous local and non-local deep learning. *Nat. Commun.* **2023,** *14* (1), 3217.
7. Moriarty, N. W.; Grosse-Kunstleve, R. W.; Adams, P. D., electronic Ligand Builder and Optimization Workbench (eLBOW): a tool for ligand coordinate and restraint generation. *Acta Crystallogr., Sect. D: Biol. Crystallogr.* **2009,** *65* (Pt 10), 1074-80.
8. Emsley, P.; Cowtan, K., Coot: model-building tools for molecular graphics. *Acta Crystallogr., Sect. D: Biol. Crystallogr.* **2004,** *60* (Pt 12 Pt 1), 2126-32.
9. Afonine, P. V.; Poon, B. K.; Read, R. J.; Sobolev, O. V.; Terwilliger, T. C.; Urzhumtsev, A.; Adams, P. D., Real-space refinement in PHENIX for cryo-EM and crystallography. *Acta Crystallogr., Sect. D: Struct. Biol.* **2018,** *74* (Pt 6), 531-544.
